# Assessing the visual and cognitive demands of in-vehicle information systems

**DOI:** 10.1186/s41235-019-0166-3

**Published:** 2019-06-21

**Authors:** David L. Strayer, Joel M. Cooper, Rachel M. Goethe, Madeleine M. McCarty, Douglas J. Getty, Francesco Biondi

**Affiliations:** 1 Department of Psychology, University of Utah, 380 S. 1530 E. RM 502, Salt Lake City, UT 84112 USA; 2 American Automobile Association, Inc, Heathrow, Florida USA; 3 Department of Kinesiology, University of Windsor, Windsor, ON Canada

**Keywords:** IVIS, In-vehicle information system, Visual demand, Cognitive demand, Driver workload

## Abstract

**Background:**

New automobiles provide a variety of features that allow motorists to perform a plethora of secondary tasks unrelated to the primary task of driving. Despite their ubiquity, surprisingly little is known about how these complex multimodal in-vehicle information systems (IVIS) interactions impact a driver’s workload.

**Results:**

The current research sought to address three interrelated questions concerning this knowledge gap: (1) Are some task types more impairing than others? (2) Are some modes of interaction more distracting than others? (3) Are IVIS interactions easier to perform in some vehicles than others? Depending on the availability of the IVIS features in each vehicle, our testing involved an assessment of up to four task types (audio entertainment, calling and dialing, text messaging, and navigation) and up to three modes of interaction (e.g., center stack, auditory vocal, and the center console). The data collected from each participant provided a measure of cognitive demand, a measure of visual/manual demand, a subjective workload measure, and a measure of the time it took to complete the different tasks. The research provides empirical evidence that the workload experienced by drivers systematically varied as a function of the different tasks, modes of interaction, and vehicles that we evaluated.

**Conclusions:**

This objective assessment suggests that many of these IVIS features are too distracting to be enabled while the vehicle is in motion. Greater consideration should be given to what interactions *should* be available to the driver when the vehicle is in motion rather than to what IVIS features and functions *could* be available to motorists.

**Electronic supplementary material:**

The online version of this article (10.1186/s41235-019-0166-3) contains supplementary material, which is available to authorized users.

## Significance

Driver distraction is increasingly recognized as a significant source of motor vehicle injuries and fatalities on the roadway. Recent technological advances allow motorists to perform many complex multimodal interactions that are unrelated to the primary task of driving. In many instances, integrated in-vehicle information systems (IVIS) are exacerbating the distracted driving problem because they support activities that are just too distracting. Our evaluations found several instances in which drivers could perform complex multimodal interactions on these information systems. For example, 65% of the vehicles we tested supported texting and 25% supported destination entry using a navigation system when the vehicle was in motion. The US National Highway Traffic Safety Administration’s visual-manual guidelines recommend against in-vehicle electronic systems that allow drivers to perform these complex and time-consuming visual-manual interactions when the vehicle is moving. Our research found that many of these features were associated with higher demand ratings. Locking out these activities and shortening the task interaction time are two methods that would reduce the overall demand on drivers and make roads safer. Greater consideration should be given to what interactions *should* be available to the driver when the vehicle is in motion rather than to what features and functions *could* be available to motorists.

## Background

New automobiles provide a number of features that allow motorists to perform a variety of secondary tasks unrelated to the primary task of driving. Many of these IVIS involve complex, multimodal interactions to perform a task. For example, to select a music option a driver might push a button on the steering wheel, issue a voice-based command, view options presented on a liquid crystal display (LCD) located in the center stack, and then select an option using the touchscreen controls on the LCD display. Complex multimodal IVIS interactions such as this may distract motorists from the primary task of driving by diverting the eyes, hands, and/or mind from the roadway (Regan, Hallett, & Gordon, 2011; Regan & Strayer, 2014).

Driver distraction arises from a combination of sources (Ranney, Garrott, & Goodman, 2000; Strayer, Watson, & Drews, 2011). Impairments to driving can be caused by a competition for visual information processing, for example when motorists take their eyes off the road to perform IVIS interactions. Impairments can also come from manual interference, as in cases where drivers take their hands off the steering wheel to perform a task. Finally, cognitive sources of distraction occur when attention is withdrawn from the processing of information necessary for the safe operation of a motor vehicle. These sources of distraction can operate independently, but they are not mutually exclusive, and therefore different IVIS interactions can result in impairments from one or more of these sources. In fact, few if any tasks are “process pure” (Jacoby, 1991) and instead often place demands on multiple resources (Wickens, 2008).

Driver distraction is caused by a diversion of attention from the primary task of operating a motor vehicle (Regan et al., 2011; Regan & Strayer, 2014) resulting in impairments to driving. In some cases, this may involve the concurrent performance of a task that is unrelated to driving (e.g., placing a cell phone call). In other cases, this may involve mis-prioritization of the component tasks associated with operating the vehicle (e.g., attending to a navigational display instead of attending to the forward roadway). It is useful to consider two theoretical accounts for why such interference occurs (e.g., Bergen, Medeiros-Ward, Wheeler, Drews, & Strayer, 2014).

On the one hand, domain-general accounts attribute dual-task interference to a competition for general computational or attentional resources that are distributed flexibly between the various tasks (e.g., Kahneman, 1973; Navon & Gopher, 1979). When two tasks require more resources than are available, performance on one or both of the tasks is impaired. This class of models suggests a transitive property of interference, such that if two tasks, A and B, exhibit dual-task interference and two tasks, B and C, exhibit dual-task interference, then the combination of tasks A and C should also exhibit dual-task interference so long as none of the tasks has reached a data limit.

On the other hand, domain-specific accounts attribute dual-task interference to competition for specific computational resources. The more similar two tasks are, in terms of specific processing resources, the greater the interference, or “code conflict” (e.g., Navon & Miller, 1987), or “crosstalk” (e.g., Pashler, 1994). In essence, two tasks that compete for the same neural hardware cannot be performed at the same time without impairments to one or both tasks. In the context of driving, for example, the visual system cannot process visual information from the forward roadway and information presented on a center stack display or heads-up display at the same time. As drivers perform different IVIS tasks, we looked for evidence of domain-general interference evidence, of domain-specific interference, and situations where both accounts would be supported.

Prior research has evaluated workload when motorists performed activities unrelated to driving. For example, the Crash Avoidance Metrics Partnership (CAMP; Angell et al., 2006) investigated the effects of twenty-two different secondary tasks requiring a combination of visual, manual and cognitive resources on driving performance. Some of the visual-manual tasks required participants to tune the radio or adjust fan speed using physical buttons located in the center console. Auditory-vocal tasks required drivers to listen to a book-on-tape or sport broadcasts and answer related questions. Distinctive driver-performance profiles suggested that task-induced driver workload was multimodal and characterized by different combinations of visual, manual, and cognitive components. In particular, relative to a baseline driving condition, visual-manual tasks were associated with a decrease in the detection of driving-related events and greater time spent glancing away from the forward roadway. By contrast, auditory-vocal tasks tended to focus the driver’s gaze on the forward roadway and resulted in better lane position maintenance - a phenomenon referred to as cognitive tunneling (see Medeiros-Ward, Cooper, & Strayer, 2014; Victor, Harbluk, & Engström, 2005).

In a series of studies, Reimer, Mehler, and colleagues (McWilliams, Reimer, Mehler, Dobres, & McAnulty, 2015; Mehler et al., 2015; Reimer et al., 2014) tested real-world infotainment systems. In Mehler et al. (2015), participants drove two vehicles (2013 Chevrolet Equinox, 2013 Volvo XC60) and interacted with the infotainment systems (MyLink and Sensus, respectively). A combination of ocular measures, subjective workload ratings, and behavioral metrics (e.g., task completion time) was adopted to examine levels of driver workload associated with completing contact calling and navigation-related tasks. Results showed that using visual-manual systems resulted in longer and more frequent off-road glances than auditory-vocal systems. Self-report measures of workload for voice interfaces were higher than those for visual-manual systems. However, the task completion time data showed mixed results, with benefits of auditory-vocal systems observed with MyLink disappearing when drivers used the Sensus system.

Our prior research provided a comprehensive assessment of cognitive workload associated with voice-based interactions, an activity known to divert attention from the driving task and lead to cognitive distraction (Strayer et al., 2015, Strayer, Cooper, Turrill, Coleman, & Hopman, 2016, 2017b). We used converging methods to provide a systematic analysis of the workload associated with different voice-based interactions. This included collecting a variety of performance measures (e.g., primary-task measures, secondary-task measures, subjective measures, and physiological measures) to provide a fine-grained assessment of variations in driver workload as they performed different tasks (e.g., calling and dialing, audio entertainment, text messaging). In Strayer et al. (2016), 257 subjects participated in a week-long evaluation of the IVIS interaction in one of 10 different model-year 2015 automobiles. After an initial assessment of the cognitive workload, participants took the vehicle home for 5 days and practiced using the system. At the end of the 5 days of practice, they returned and the workload of these IVIS interactions was reassessed. The cognitive workload was found to be moderate to high and was associated with the intuitiveness and complexity of the system and the time it took participants to complete the interaction. Importantly, practice did not eliminate the interference. In fact, interactions that were difficult on the first day were still relatively difficult to perform after a week of practice. There were also long-lasting residual costs after the IVIS interactions had terminated. We suggested that the higher levels of workload should serve as a caution because these voice-based interactions can be cognitively demanding and ought not to be used indiscriminately while operating a motor vehicle.

Task duration is central to the issue of workload assessment. A simple but elegant argument for the importance of task duration has been outlined by Shutko and Tijerina (2006). They suggest that evaluation of task duration is critical not because it reflects a cumulative effect of load, but because it represents the time over which an unexpected event might occur. Using a simple exposure-based model, they argue that all else being equal, a task that takes twice as long to complete will result in twice the potential risk of an adverse event. Other models suggest a cascading negative effect of task duration on situation awareness (e.g., Fisher & Strayer, 2014; Strayer & Fisher, 2016).

There is no clear consensus on what constitutes an acceptable interaction time for a secondary task. Problematically, the issue is confounded by research suggesting that secondary tasks are often sensitive to whether testing is completed in a static (i.e., not driving) or dynamic (i.e., driving) environment (Young et al., 2005), the age of participants (McWilliams, Reimer, Mehler, Dobres, & Coughlin, 2015), and performance characteristics of the primary or secondary tasks (Tsimhoni, Yoo, & Green, 1999). Because of the visual demands associated with driving, visual secondary tasks generally take longer to complete when performed concurrently with driving. Additionally, due to natural aging processes, older adults generally take longer to perform tasks than younger adults. These issues aside, a number of organizations have provided guidance on what constitutes an acceptable secondary task duration (e.g., Driver Focus-Telematics Working Group, 2006; Japan Automobile Manufacturers Association, 2004; National Highway Traffic Safety Administration, 2013).

For example, National Highway Traffic Safety Administration (NHTSA) (2013) has issued a set of voluntary guidelines for visual/manual tasks that suggest that tasks should require no more than 12 s of total eyes off road time (TEORT) to complete. This 12-s rule is based on the societally acceptable risk associated with tuning an analog in-car radio. Using visual occlusion, a method specified by NHTSA to evaluate visual-manual tasks, motorists can view the driving environment for 12 s and vision is occluded for 12 s in 1.5-s on/off intervals. When assessed with the visual occlusion methodology, the NHTSA guidelines provide an implicit maximum of 24 s of total task time (i.e., 12 s of shutter open time + 12 s of shutter closed time for a total task time of 24 s). While intended for visual/manual tasks, these guidelines provide a reasonable upper limit for multimodal task durations of any type.

An important prerequisite for duration-based measures of secondary task performance is the definition of a task. We use the definition provided by Burns, Harbluk, Foley, and Angell (2010), which is a derived from the Alliance of Automobile Manufactures, International Standards Organization (ISO), and JAMA guidelines. Burns et al., suggest that a task can be defined as a sequence of inputs leading to a goal at which the driver will normally persist until the goal is reached. However, we differentiate between continuous and discrete tasks that are shaped by different performance goals. Fundamental to secondary discrete tasks is a performance goal with a finite beginning and end state (e.g., changing the audio source, dialing a phone number, calling a contact, entering a destination into a navigation unit, etc.). Conversely, continuous tasks are characterized by performance maintenance over an indefinite period of time, often with no clear termination state (Schmidt & Lee, 2005) (e.g., conversing via a cell phone, listening to music, following route guidance, etc.). Given the nature of discrete tasks, a failure to account for task duration during assessment provides an incomplete picture of distraction potential.

### Research questions

An important knowledge gap concerns the workload associated with making complex multimodal IVIS interactions. What are the visual and cognitive demands associated with different modes of IVIS interactions (e.g., auditory/vocal interactions versus visual/manual interactions)? To what degree do the different IVIS task types (e.g., audio entertainment, calling and dialing, text messaging, navigation, etc.) place differential demands on visual and cognitive resources? Vehicles clearly differ in their configuration and layout, but do they differ in the visual and cognitive demands of IVIS interactions? Are there tradeoffs for IVIS interactions performed with one task or mode of interaction versus another? For example, auditory/vocal inputs may have lower levels of visual demand than issuing commands using a visual/manual touchscreen, but the time taken to perform the interaction may be longer in the former than the latter. Surprisingly little is known about how these complex multimodal IVIS interactions impact the driver’s workload. Given the ubiquity of these systems, the current research sought to address three interrelated questions concerning this knowledge gap.

First, are some task types more impairing than others? The IVIS interactions support a variety of secondary tasks that are unrelated to the primary task of driving. Some of these interactions may be considered to be sufficiently impairing that they are locked out by the automaker when the vehicle is in motion (e.g., social media interactions are locked out by most automakers). However, not all secondary tasks are equivalent in distraction potential (e.g., Strayer et al., 2015). They differ in terms of task goals (e.g., play a song, send a text, place a call, etc.). Tasks differ in duration, ranging from a few seconds to a few minutes to complete, with greater distraction potential associated with greater task duration (e.g., Burns et al., 2010). Tasks differ in the way that they are implemented and they may be performed using different modes of interaction (i.e., tasks may be easier to perform using one mode of interaction than another). Tasks may also be performed using a streamlined “one-shot” interaction, or via a series of interactive steps. The current research assessed which task types were most distracting. It is possible that some tasks may be too demanding to be enabled when the vehicle is in motion, regardless of the mode of interaction.

Second, are some modes of interaction more distracting than others? In many instances, a task can be performed using auditory/vocal commands, visual/manual interactions, or, as in the example discussed above, a hybrid combination of both auditory/vocal and visual/manual interactions. If the workload associated with one mode of interaction differs from another, the differences may be offset by the time it takes to perform the interaction. For example, a visual/manual touchscreen interaction may divert the driver’s eyes from the roadway while an auditory/vocal interaction may keep the eyes on the road; however, if the time to perform an auditory/vocal interaction takes longer than the visual/manual interaction, any benefits of the former may not be realized. Moreover, just because auditory/vocal interactions tend to keep the eyes on the road does not provide a guarantee that drivers will see what they are looking at (Strayer, Drews, & Johnston, 2003; Strayer & Fisher, 2016). The current research is designed to provide an objective benchmark for the level of distraction caused by different modes of IVIS interaction.

Third, are IVIS interactions easier to perform in some vehicles than others? A trip to the automobile dealer’s showroom will quickly illustrate that vehicles differ in the features, functions, and type of human-machine interface of the IVIS. Are these differences in the IVIS merely cosmetic, or do the differences result in differential workload to perform the same IVIS functions? Vehicles differ in the number and complexity of button interactions on the steering wheel, the size, resolution, and functions supported on the center stack LCD, manual buttons on the center stack and their configuration, and the other unique modes of interaction (e.g., heads-up displays, gesture controls, rotary dials, writing pads, etc.). Moreover, vehicles often provide more than one way to perform a task. There are often cross-modal interactions wherein the task is initiated using one mode of interaction (e.g., voice commands), and then transitions to another mode of interaction (e.g., touchscreen interactions). Some IVIS interactions are ubiquitous (e.g., calling and dialing and audio entertainment), whereas others are supported by one automaker but not another (e.g., destination entry for a navigation system while the vehicle is in motion). The current research compared the IVIS interactions supported by different automakers to determine if they differ in the workload associated with their use. If there are differences in the overall demand of the IVIS interactions, what are the bases for the differences?

### Experimental overview

Our prior research found that it was necessary for the driver to be driving the vehicle in order to accurately assess the concurrent workload associated with IVIS interactions - that is, dynamic testing rather than static testing (cf., SAE J2365, 2016). This was true for IVIS interactions with high levels of cognitive demand, such as using voice commands to interact with the IVIS (e.g., Strayer et al., 2015, Strayer et al., 2016, 2017b). With cognitive demand, the task of driving added a constant increase to the estimates of driver workload (e.g., the time to perform a purely voice-based IVIS interaction in a moving vehicle was increased by a constant from the time to perform the same interaction in a stationary vehicle).^1^ This problem was exacerbated for IVIS interactions with high levels of visual demand, such as making selections on a center stack touchscreen, where the time to perform an IVIS interaction in a moving vehicle was an increasing linear function of the time to perform the same interaction in a stationary vehicle. Consequently, all estimates of driver workload in the current research were obtained when participants were driving the vehicle and engaged in IVIS interactions or driving in one of the control conditions (i.e., a dynamic testing method). The driving route we used was a low-density residential section of roadway with a speed limit of 25 MPH, chosen due to the relatively modest driving demands imposed by the roadway.

To properly scale the driver’s workload while interacting with the IVIS, several control conditions were required. First, a single-task driving baseline was needed to estimate the workload of the driver when they were driving the vehicle without the additional workload imposed by the IVIS interactions. This single-task baseline controls for any differences between participants and the workload associated with driving the different vehicles. The single-task baseline anchors the low end of the cognitive and visual workload estimates derived in our research.

To scale cognitive demand, a high workload cognitive task was selected that could be performed in the same way by all participants in all vehicles. The high workload referent task we used was an N-back task (e.g., Mehler, Reimer, & Dusek, 2011; Zhang, Angell, Pala, & Shimonomoto, 2015) in which a pre-recorded series of numbers ranging from 0 to 9 were presented at a rate of one digit every 2.25 s. Participants were instructed to say out loud the number that was presented two trials earlier in the sequence. The N-back task places a high level of cognitive demand on the driver without imposing any visual demands. Using the single-task baseline and N-back referent, provided a way to standardize the cognitive demand of the different IVIS interactions. That is, after controlling for any differences in workload associated with different vehicles using the single-task baseline, IVIS interactions can be directly compared to the N-back task to provide an objective measure of cognitive demand associated with their performance.

**Fig. 1:**
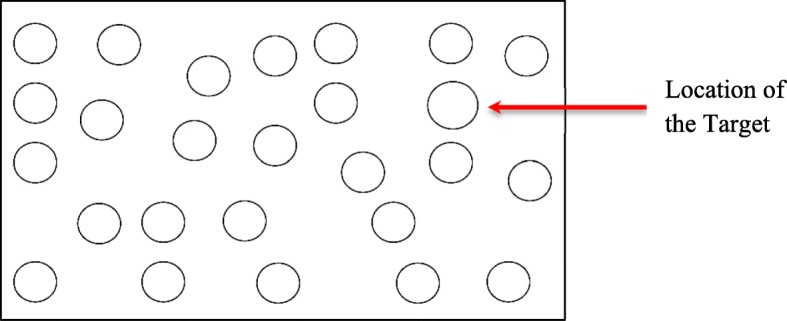
An example of the surrogate reference task (SuRT) task that required participants to touch the location of the target circle

To scale visual demand of the IVIS interactions, a high workload visual referent task was selected that could be performed in the same way by all participants in all vehicles. The high workload task we used was a variant of the ISO TS 14198 Surrogate Reference Task (SuRT; Engström & Markkula, 2007; Mattes, Föhl, & Schindhelm, 2007, Zhang et al., 2015) that required participants to use their finger to touch the location of target items (larger circles) presented in a field of distractors (smaller circles) on an iPad Mini tablet computer that was mounted in a similar position in all the vehicles. Immediately after touching the location of the target, a new display was presented with a different configuration of targets and distractors. The trial sequence would not advance until the correct location was touched on the screen. The SuRT task, illustrated in Fig. 1, is a based on a feature search (e.g., Treisman & Gelade, 1980) for the size of the larger circle and the participant’s response is to identify the location of the target (as opposed to a present/absent response).

Drivers were instructed to perform the SuRT as a secondary task while giving the driving task highest priority. The SuRT task places a high level of visual demand on the driver because they must look at the display in order to locate the targets and then touch the display to indicate their response. Using the single-task baseline and SuRT referent provides a way to standardize the visual demand of the different IVIS interactions. That is, after controlling for any differences in workload associated with different vehicles using the single-task baseline, IVIS interactions can be directly compared to the SuRT task to provide an objective measure of visual demand associated with its performance.

The N-back referent task induces a high level of cognitive demand and does not present any visual information for the driver to look at. However, it is well-known that high levels of cognitive demand often alter the visual scanning behavior of the driver (e.g., see Strayer & Fisher, 2016 for a review). That is, the N-back task may impair what the driver sees. Similarly, the SuRT referent induces a high level of visual demand by requiring the driver to look at a touchscreen to locate a target amongst distractors. However, in addition to taking the driver’s eyes off the roadway to perform the task, visual attention is required to perform the SuRT task. Pilot testing of the SuRT task found a visual search slope of approximately 20 msec/item, a value above the upper threshold associated with automatic visual search (e.g., Schneider & Shiffrin, 1977; Shiffrin & Schneider, 1977). Thus, the SuRT task has high visual/manual demand and modest cognitive demand.

The current research used converging performance measures to benchmark the workload of the IVIS interactions. This included the collection of *subjective* estimates from the driver on their workload using the NASA-Task Load Index (Hart & Staveland, 1988) at the end of testing each IVIS interaction.

We also assessed driver workload using the Detection Response Task (DRT), an ISO protocol for measuring attentional effects of cognitive load (ISO 17488, 2015). The DRT procedure involves presenting a simple stimulus (e.g., a changing light or vibrating buzzer) every 3–5 s and requiring the driver to respond to these events when they detect them by pressing a microswitch (button) was attached to the driver’s left thumb so that the button could be depressed against the steering wheel when participants detected the vibration (or light). That is, the DRT is a simple response task (RT) that is performed concurrently with other activities (e.g., driving). As the workload of driving and/or the IVIS interactions increase, the reaction time to the DRT stimulus increases and the likelihood of detection of the DRT stimulus (i.e., the hit rate) decreases (e.g., Strayer et al., 2015, Strayer et al., 2016, 2017b). The DRT has proven to be very sensitive to dynamic changes in the driver’s workload (e.g., Strayer et al., 2017a). The DRT provides an *objective* assessment of the driver’s workload associated with different IVIS interactions, with minimal interference in performance of the driving task (see Strayer et al., 2013, Castro, Cooper, & Strayer, 2016, Palada, Strayer, Neal, Ballard, & Heathcote, 2017).

We used two variants of the DRT in our research. The first variant was a vibrotactile DRT, in which a vibrating buzzer, that feels similar to a vibrating smartphone, was attached to the participant’s left collarbone and a microswitch was attached to a finger on the driver’s left hand so that it could be depressed against the steering wheel when they detected the vibration. The vibrotactile DRT provides a sensitive measure of the participant’s cognitive load as they perform different IVIS interactions. As the cognitive demand increases, the RT to the vibrotactile DRT increases. These RT differences were calibrated using the single-task baseline and N-back referent to anchor the workload of the IVIS interactions in different vehicles.

Specifically, evaluation of the cognitive demand of any IVIS interaction involved an initial subtraction from any differences between vehicles and/or participants obtained in the single-task baseline (i.e., this defined the relative demand associated with an IVIS interaction). This relative cognitive demand was compared to the N-back task (i.e., the difference between the N-back task and single-task baseline defined the relative cognitive demand of the N-back task). The Cognitive Demand Ratio (CDR) was defined as the ratio of the relative cognitive demand of an IVIS interaction to the relative cognitive demand associated with the N-back task.

The CDR provides a standardized metric for comparison across IVIS interactions (both within a vehicle and between vehicles). For example, if an IVIS interaction has a CDR that is between 0 and 1, the cognitive demand of that interaction is greater than the single-task baseline and less than the N-back task. If an IVIS interaction has a CDR greater than 1, then the cognitive demand of that IVIS interaction exceeds the N-back task. Furthermore, if the CDR of an IVIS interaction in one vehicle is greater than the same IVIS interaction in another vehicle, the two vehicles differ in the cognitive demand of that interaction, with the former being greater than the latter.

The second variant of the DRT used a light that was projected onto the windshield in the driver’s line of sight as they looked at the forward roadway. When the DRT light changed from orange to red, the participant was instructed to press the microswitch attached to their finger when they detected the changing light (the same response that was used for the vibrotactile DRT). The visual DRT provides a sensitive measure of the participant’s visual load as they perform different IVIS interactions. As the visual demand increases, the detection of the changing light decreases (i.e., a decrease in hit rate). These hit rate differences were calibrated using the single-task baseline and SuRT task to anchor the workload of the IVIS interactions in different vehicles.

Evaluation of the visual demand of any IVIS interaction involved an initial subtraction from any differences between vehicles and/or participants obtained in the single-task baseline (i.e., this defined the relative visual demand associated with an IVIS interaction). This relative visual demand was compared to the SuRT task (i.e., the difference between the SuRT referent and single-task baseline defined the relative visual demand of the SuRT task). The visual demand ratio (VDR) was defined as the ratio of the relative visual demand of an IVIS interaction to the relative visual demand associated with the SuRT task.

As with CDR, VDR provides a standardized metric for comparison across IVIS interactions (both within a vehicle and between vehicles). For example, if an IVIS interaction has a VDR that is between 0 and 1, the visual demand of that interaction is greater than the single-task baseline and less than the SuRT task. If an IVIS interaction has a VDR greater than 1, then the visual demand of that IVIS interaction exceeds the SuRT task. Furthermore, if the VDR of an IVIS interaction in one vehicle is greater than the same IVIS interaction in another vehicle, the two vehicles differ in the visual demand of that interaction, with the former being greater than the latter.

In order to capture the effects of task duration, our measures of momentary cognitive, visual, and subjective task demand were combined into a metric of overall demand and scaled by task completion time. Tasks that took longer than 24 s resulted in an upward biasing of overall demand whereas tasks that took less than 24 s resulted in a downward bias. Of the metrics that fed into the overall workload metric, total task time may be most amenable to modification through design. Our investigation found that factors such as menu depth, display clutter, system responsivity, dialog verbosity, cellular connection stability, and server performance all play a significant role in task duration (e.g., Biondi, Getty, Cooper, & Strayer, 2018). The time required for a user to complete a task can be reduced through the careful performance evaluation, resulting in a reduction in exposure duration.

## Method

### Participants

After approval from the University of Utah Institutional Review Board (IRB (number 00052567)), 120 participants (54 female), with an age range of 21–36 years (mean (M) = 25 years) and a reported average of 9.1 driving hours per week, were recruited via flyers and social media. All participants were native English speakers, had normal or corrected-to-normal vision, held a valid driver’s license and proof of car insurance, and had not been the at-fault driver in an accident within the past 2 years. Compensation was prorated at US$20 per hour. Prior to participation, a Motor Vehicle Record report was obtained by the University of Utah’s Division of Risk Management to ensure a clean driving history. Each participant was also required to complete a 20-min online defensive driving course and pass the accompanying certification test, as per University of Utah policy.

A total of 24 participants were tested in each vehicle. The duration of a testing session for a vehicle was dependent on the features and functions available in each vehicle (testing ranged from 2.5 to 3.5 h). Participants were initially naïve to the specific IVIS tasks and systems but were trained until they felt comfortable performing each of the requested interactions. Additionally, participants gained broad experience with the different systems, tasks, and modes of interaction offered by each vehicle through repeated research participation.

### Equipment

**Fig. 2:**
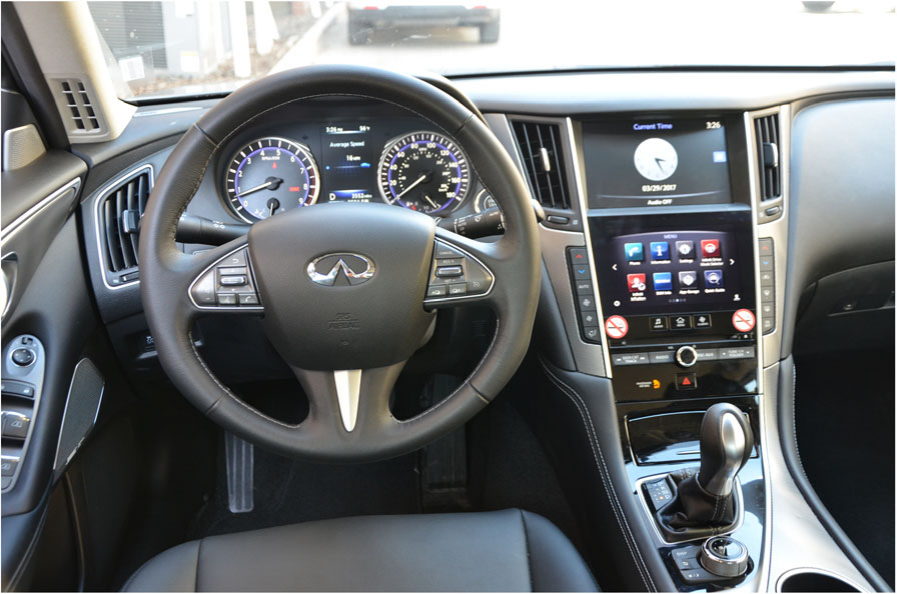
The interior of a 2017 Infinity Q50 Premium. Note dashboard display, two center stack displays, and the buttons on the steering wheel, center stack, and rotary dial on the center console

The vehicles used in the study are listed in Table 1. Vehicles were selected for inclusion in the study based on an initial assessment of market share of the vehicle, the IVIS features available in the vehicle, and availability of vehicles for testing. An example of a feature-rich IVIS is presented in Fig. 2. Vehicles were acquired through Enterprise rental car, short-term leases from automotive dealerships, or purchased for testing. This sample was representative of 30% of the market share in North America. Obviously, the specific sequence of actions required to perform the different tasks varied as a function of original equipment manufacturer (OEM) and modality of interaction.

**Table 1:** Vehicles used in the study

• 2017 Audi Q7 Premium Plus	
• 2018 BMW 430i	
• 2017 Buick Enclave	
• 2017 Cadillac XT5 Luxury	
• 2017 Chevrolet Equinox LT	
• 2018 Chevrolet Silverado LT	
• 2017 Chevrolet Traverse LT	
• 2017 Chrysler 300 C	
• 2017 Dodge Durango GT	
• 2017 Ford F250 XLT	
• 2017 Ford Fusion Titanium	
• 2017 Ford Mustang GT	
• 2017 GMC Yukon SLT	
• 2017 Honda Civic Touring	
• 2017 Honda Ridgeline RTL-E	
• 2017 Hyundai Santa Fe Sport	
• 2017 Hyundai Sonata Base	
• 2017 Infiniti Q50 Premium	
• 2017 Jeep Compass Sport	
• 2017 Jeep Grand Cherokee Limited	
• 2018 Kia Optima LX	
• 2017 Kia Sorento LX	
• 2017 Kia Sportage LX	
• 2017 Land Rover Range Rover Sport	
• 2017 Lincoln MKC Premiere	
• 2017 Mazda 3 Touring	
• 2017 Mercedes C300	
• 2017 Nissan Armada SV	
• 2017 Nissan Maxima SV	
• 2017 Nissan Rogue SV	
• 2017 Ram 1500 Express	
• 2018 Ram 1500 Laramie	
• 2017 Subaru Crosstrek Premium	
• 2017 Tesla Model S 75	
• 2017 Toyota Camry SE	
• 2017 Toyota Corolla SE	
• 2017 Toyota RAV4 XLE	
• 2017 Toyota Sienna XLE	
• 2017 Volkswagen Jett S	
• 2017 Volvo XC60 T5 Inscription

Identical LG K7 android phones on the T-Mobile mobile network were paired via Bluetooth with each vehicle. Each vehicle was also equipped with two Garmin VirbXE action cameras, one mounted under the rear-view mirror to provide recordings of participants’ faces, and an additional camera mounted near the passenger seat shoulder to provide a view of the dash area for infotainment interaction. Video was recorded at 30 frames per second, at 720-p resolution. An iPad Mini 4 (20.1 cm diagonal LED-backlit Multi-Touch display) was connected to each vehicle via USB and was pre-loaded with a small music library. Identical Acer R11 laptop computers were utilized for data collection in the vehicle.

### Stimuli

Participants completed tasks requiring IVIS interaction. Depending on the vehicle, participants would interact with the system to perform tasks with audio entertainment, calling and/or dialing, navigation, and text messaging. Also dependent on vehicle interface was the method by which participants would interact (see Additional file 1 for complete detail of the tasks performed in each vehicle). All vehicles had voice recognition, however the vehicles differed on visual/manual interaction (e.g., touchscreen, manual buttons, rotary wheel, and wheel pad). The interaction tasks in each vehicle were matched as closely as possible given the differences in the systems’ capabilities.

### DRT

**Fig. 3:**
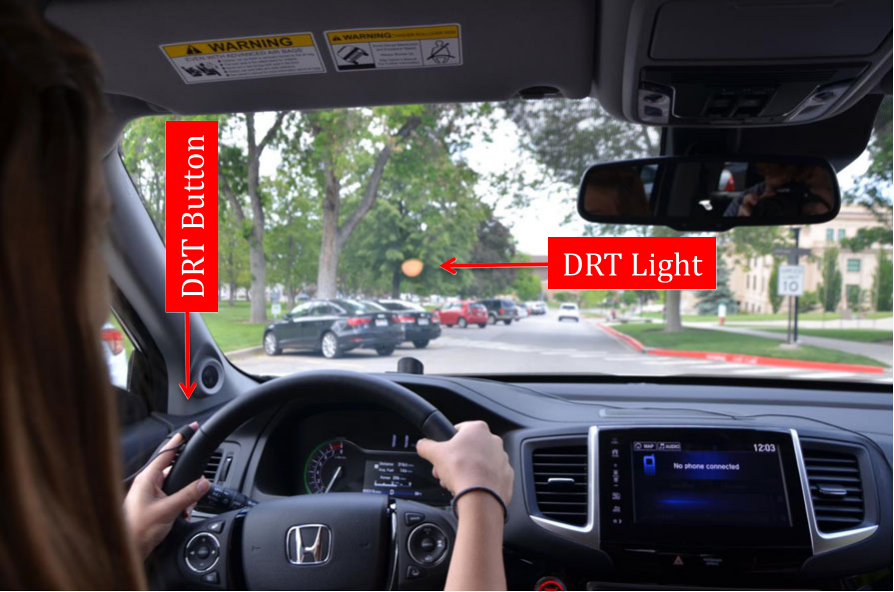
A research participant driving the 2017 Honda Ridgeline. Note the orange detection response task (DRT) light projected onto the windshield in the driver’s forward field of view and the DRT microswitch attached to the participant’s left index finger. The vibrotactile device attached to the participant’s collarbone is not shown in the photo

Participants were required to respond to a vibrotactile and visual DRT as per ISO 17488 (2015). A vibrotactile device was placed on the participant’s left collarbone area and a microswitch was attached to either the index or middle finger of the left hand so that it could be depressed against the steering wheel. A visual DRT light was placed along a strip of Velcro on the dashboard in such a way that the participant could not directly gaze upon the light but instead saw the reflection in the windshield directly in their line of sight (Castro et al., 2016; Cooper, Castro, & Strayer, 2016). An example of the DRT configuration is presented in Fig. 3. Millisecond resolution response time to the vibrotactile onset or LED light was recorded via an embedded micro-controller and stored on the host computer.

Following the ISO guidelines (2015), the vibrotactile device emitted a small vibration stimulus, similar to a vibrating cell phone. The LED light stimulus was a change in color from orange to red. These changes cued the participant to respond as quickly as possible by pressing the microswitch against the steering wheel. The tactor and light were equiprobable and programmed to occur every 3–5 s (i.e., a rectangular distribution of inter-stimulus intervals between 3 and 5 s) and lasted for 1 s or until the participant pressed the microswitch. The task of driving was considered the primary task, the interaction with the IVIS was considered the secondary task, and responding to the DRT was considered a tertiary task.

### Procedure

Participants completed tasks involving interacting with the infotainment system in the vehicle to achieve a particular goal (i.e., using the touchscreen to tune the radio to a particular station, using voice recognition to find a particular navigation destination, etc.) while driving. Tasks were categorized into one of four task types: audio entertainment, calling and dialing, text messaging, and navigation, depending on vehicle capabilities. These task types were completed via different modalities equipped in each vehicle (i.e. touchscreen, voice recognition, rotary wheel, draw pad, etc.) for each interaction. The order of interactions was counterbalanced across participants.

The possible task types performed by the participant are listed subsequently. The specific syntax and command sequence to perform the different tasks in each of the vehicles and modalities of interaction are provided in Additional file 1.

- Audio entertainment: participants changed the music to different FM and AM stations, a satellite radio source, the LG K7 phone connected via Bluetooth, and the Mini iPad connected via USB:
- Calling and dialing: a list of 91 contacts with a mobile and/or work number was created for participant use. In vehicles capable of dialing phone numbers, participants were instructed to dial the phone number 801–555-1234 and their own phone number.
- Text messaging: depending on the texting capabilities of each vehicle, participants either listened to short text messages sent by other LG K7 phones or sent a new text from the list of predetermined messages specific to each vehicle.
- Navigation: participants started and canceled route guidance to different local and national businesses that differed according to the options presented by each system.

The potential modes of interaction performed by the participant are listed subsequently. Interaction modalities were selected and individual tasks created based on vehicle capabilities:

- Center stack: the center stack is located in the center of the dash to the right of the driver. A visual display is used to present textual and/or graphical information. Center stack systems often include a touchscreen interface to support visual/manual interactions so that drivers can select an option and navigate menus by touch and/or use slider bars to scroll through options displayed on the screen. With some vehicles, the selection of options may be made with manual buttons surrounding the touchscreen.

- Auditory vocal: a voice-based interaction is initiated by the press of a physical button on the steering wheel or center stack. Microphones installed in the vehicle pick up the driver’s voice commands and process them to perform specific functions and access help menus in the vehicle. Possible voice command options may be presented aurally or displayed on the vehicle’s center stack to aid the driver in making valid commands.
- Center console: the center console is located between the driver and passenger front seats. The interactions are made through a rotary dial that allows drivers to scroll through menu items presented on the center stack visual display. Another interaction variant uses a writing pad where drivers use their finger to write out commands.

### Driving route

**Fig. 4:**
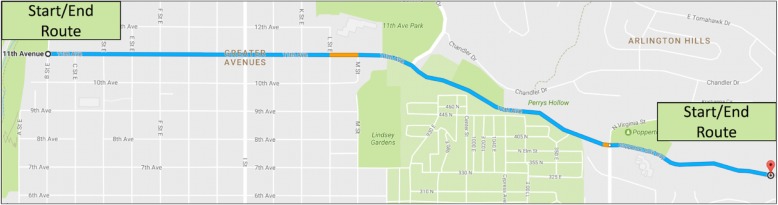
A bird’s-eye view of the driving route used in the study

A low-traffic residential road with a 25-mph speed limit was used for the on-road assessment. The route, depicted in Fig. 4, contained four stop signs and two speed bumps. The participants were required to follow all traffic laws and adhere to the 25-mph speed limit at all times. The length of road was approximately 2 miles one-way with an average drive time of 6 min in each direction. A researcher was present in the passenger seat of each vehicle for safety monitoring and data collection.

### Training

Before the study commenced, participants were given time to adjust and familiarize themselves with the vehicle while driving a practice run on the designated route. During the familiarization drive, the researcher pointed out potential road hazards. After participants felt comfortable in the vehicle, they were trained on how to respond to the DRT. The researcher verified that participants responded appropriately to 10 stimuli presented between 3 and 5 s apart and that they had response times of less than 500 ms. Next, they were trained on how to interact with and complete tasks via a particular modality before each condition began. In order to be considered properly trained, participants were required to perform three trials without error immediately before the testing commenced for each of the IVIS interactions. Once participants expressed confidence in their ability to interact with the system, the experimental run began.

Participants were instructed to drive the designated route from one end to another, performing the IVIS interactions as instructed by the experimenter several times on each drive. When the participant reached the end of the route, they were instructed to pull over, marking the end of one of the experimental blocks. The next experimental block began in the opposite direction on the designated route, and this process was repeated until all conditions had been completed.

While driving, verbal task instructions provided by the researcher were given to the participant (e.g., “Using the touchscreen, tune radio to 96.3 FM”). A complete list of the tasks performed in each vehicle is presented in Additional file 1. Participants were instructed to not initiate the task until the researcher told them to do so by saying, “Go.” Once the given task was complete, the participant would say, “Done.” The researcher would mark the task start and end time of each task by depressing a key on the data collection computer for later analysis of association with timing of on-task performance. DRT trials were considered valid for statistical analysis if they fell between these start and end times. Participants were allowed to take as much time as needed to complete each task. A minimum 10-s interval was provided between tasks. The total number of tasks in each 2-mile run varied with task duration, ranging from 5 to 11. Participants also performed three control tasks while driving the designated route. The control tasks were:

- Single-task baseline: participants performed a single-task baseline drive using the vehicle being tested on the designated route without interacting with the IVIS. During the single-task baseline, participants responded to the DRT stimuli.

- Auditory N-back task: the auditory N-back task presented a pre-recorded series of numbers at a rate of one digit every 2.25 s. Participants listened to auditory lists of numbers ranging from 0 to 9 presented in a randomized order. They were instructed to say out loud the number that was presented two trials earlier in the sequence. Participants were instructed to respond as accurately as possible to the N-back stimuli and the research assistant monitored performance in real time. During the auditory N-back task, participants also responded to the DRT stimuli.
- SuRT task: the variant of the SuRT task used in this research presented a target on the display with 21–27 distractors. The target was an open circle 1.5 cm in diameter and the distractors were open circles 1.2 cm in diameter. The SuRT task was presented on an iPad Mini 4 with circles printed in black on a white background. The participant’s task was to touch the location of the target. Immediately thereafter, a new display was presented with a different configuration of targets and distractors. The location of targets and distractors was randomized across the trials in the SuRT task. Participants were instructed to continuously perform the SuRT task while giving the driving task highest priority and the research assistant monitored performance in real-time. A research assistant instructed participants to pause the SuRT task at intersections or if there were potential hazards on the roadway. During the SuRT task, participants also responded to the DRT stimuli.

After the completion of each condition, participants were given a NASA-TLX (Hart & Staveland, 1988) to assess the subjective workload of that car’s system.

### Dependent measures

DRT data were cleaned following procedures specified in ISO 17488 (2015). Consistent with the standard, all responses briefer than 100 ms (0.6% of the total trials) or greater than 2500 ms (1.4% of the total trials) were rejected for calculations of reaction time. Non-responses or responses that occurred later than 2.5 s from the stimulus onset were coded as misses. During testing of the IVIS interactions, on-task engagement was recorded by the researcher through a key press on the DRT host computer, which allowed the identification of segments of the IVIS condition when the participant was actively engaged in an activity or had finished that activity and was operating the vehicle without IVIS interactions. Incomplete, interrupted, or otherwise invalid tasks, were marked with a key-flag and excluded from analysis. In addition, task time data were cleaned to remove any recorded tasks with a duration shorter than 3 s, which resulted in the removal of less than 0.3% of tasks. The dependent measures obtained in the study are listed below:

- DRT - reaction time: defined as the sum of all valid reaction times to the DRT task divided by the number of valid reaction times.
- DRT - hit rate: defined as the number of valid responses divided by the total number of valid stimuli presented during each condition.

Following each drive, participants were asked to fill out a brief questionnaire that posed eight questions related to the just completed task. The first six of these questions were from the NASA TLX; the final two assessed the intuitiveness and complexity of the IVIS interactions:

- Subjective measures - defined as the response on a 21-point scale for each question:
  - Mental – How mentally demanding was the task?
  - Physical – How physically demanding was the task?
  - Temporal – How hurried or rushed was the pace of the task?
  - Performance – How successful were you in accomplishing what you were asked to do?
  - Effort – How hard did you have to work to accomplish your level of performance?
  - Frustration – How insecure, discouraged, irritated, stressed, and annoyed were you?
  - Intuitiveness – How intuitive, usable, and easy was it to use the system?
  - Complexity – How complex, difficult, and confusing was it to use the system?

Task interaction time was obtained from the time stamp on the DRT host computer. The research assistant pressed a key on the keyboard on the DRT host computer to mark different activities in the real-time DRT record (e.g., on-task IVIS interactions). Task interaction time was defined as the time from the moment participants first initiated an action (i.e., when the research assistant told the participant to start a procedure) to the time when that action had terminated and the participant said, “Done”. In addition to marking on-task IVIS intervals, the research assistant also used the keyboard to identify segments of the drive when participants had to stop at intersections, interact with traffic, etc. Only the DRT trials with a stimulus onset that occurred during the on-task interval were used in the analyses reported below.

### Data analysis and modeling

The DRT data were used to provide empirical estimates of the cognitive and visual demand of the different conditions. For an estimate of cognitive demand, the average RT to the vibrotactile DRT for each participant was computed for the single-task baseline condition and for the N-back task; Eq. 1 was used to standardize the vibrotactile DRT data:

1 $$ Cognitive\ Demand=\frac{IVIS\ Task- Single\ Task}{Nback\ Task- Single\ Task} $$

Using Eq. 1, the single-task baseline would receive a rating of 0.0 and the N-back task would receive a score of 1.0. IVIS tasks tested in the vehicle were similarly scaled such that values below 1.0 would represent a cognitive demand lower than the N-back task and values greater than 1.0 would denote conditions with a higher cognitive demand than the N-back task. Note that the cognitive demand is a continuous measure ranging from 0 to ∞, with higher values indicating higher levels of cognitive demand.

To provide a concrete example, suppose a researcher is interested in determining the cognitive demand associated with using voice commands to generate and send a text message. The increase in demand associated with sending a text, relative to the single-task baseline is the numerator in Eq. 1 (Texting Task – Single Task). The numerator is scaled by the referent task and the single-task baseline (i.e., Nback Task – Single Task) to provide a demand score that ranges from 0 (i.e., no more demanding than the single task) to ∞. A score of 1.0 would indicate that sending a text message was equivalent in demand to the N-back task.

For an estimate of visual demand, the average hit rate to the visual DRT for each participant was computed for the single-task baseline condition and for the SuRT task; Eq. 2 was used to standardize the data collected from the visual DRT:

2 $$ Visual\ Demand=\frac{Single\ Task- IVIS\ Task}{Single\ Task- SuRT\ Task} $$

Using Eq. 2, the single-task baseline would receive a rating of 0.0 and the SuRT task would receive a score of 1.0. IVIS tasks tested in the vehicle were similarly scaled such that values below 1.0 would represent visual demand lower than the SuRT task and values greater than 1.0 would denote conditions with visual demand higher than the SuRT task. As with cognitive demand, the visual demand is a continuous measure ranging from 0 to ∞, with higher values indicating higher levels of visual demand.

For an estimate of subjective demand, the average of the six National Aeronautics and Space Administration (NASA) Task Load Index (TLX) ratings for each participant were computed for the single-task baseline condition and for the N-back and SuRT tasks; Eq. 3 was used to standardize the subjective estimates.

3 $$ Subjective\ Demand=\frac{IVIS\ Task- Single\ Task}{\left(\frac{Nback\ Task+ SuRT\ Task}{2}\right)- Single\ Task} $$

Using Eq. 3, the single-task baseline would receive a rating of 0.0 and average of the N-back and SuRT tasks would receive a score of 1.0. IVIS tasks tested in the vehicle were similarly scaled such that values below 1.0 would represent a subjective demand lower than the average of the N-back and SuRT tasks and values greater than 1.0 would denote conditions with subjective demand higher than the average of the N-back and SuRT tasks. As with cognitive demand, the subjective demand is a continuous measure ranging from 0 to ∞, with higher values indicating higher levels of subjective demand.

Equation 4 was used to standardize the IVIS interaction time data using the 24-s interaction time referent (National Highway Traffic Safety Administration, 2013):

4 $$ Interaction\ Time=\frac{IVIS\ Task}{24\  seconds} $$

Using Eq. 4, a task interaction time of 24 s would receive a score of 1.0. IVIS interactions tested in the vehicle were scaled such that values below 1.0 would represent a task interaction time lower than 24 s and values greater than 1.0 would denote conditions with a task interaction time greater than 24 s. The time-on-task metric is a continuous measure ranging from 0 to ∞, with higher values indicating longer task interaction time.

The 24-s task interaction referent is derived from National Highway Traffic Safety Administration (2013). Performance on the high visual/manual demand SuRT for 24 s, a score of 1.0 in our rating system, matches the NHTSA acceptable limit for total task time using the visual occlusion testing procedure. The general principle is that these multimodal IVIS interactions should be able to be performed in 24 s or less when paired with the task of operating a moving motor vehicle.

An overall workload rating was determined by combining the cognitive, visual, and subjective demand with the interaction time rating using Eq. 5. Using Eq. 5, overall demand is a continuous measure ranging from 0 to ∞, with higher values indicating higher levels of workload:

5 $$ Overall\ Demand=\frac{\left( Cognitive+ Visual+ Subjective\right)\ }{3}\ast Interaction\ Time $$

Application of these formulae provide stable workload ratings with useful performance criteria that are grounded in industry standard tasks. On occasion, however, the approach can return extreme values when either the numerator is unusually small or the task time unusually long. In order to mitigate the potential for unusual scores to skew the overall rating, scores greater than 3.5 standard deviations from the mean (< 1% of the data) were excluded from analysis.

### Experimental design

The experimental design was a 4 (task type) × 3 (modality of interaction) × 40 (vehicle) factorial with 24 participants evaluated in each vehicle. However, not all vehicles offered the full factorial design (i.e., the task type by modality of interaction factorial was not always available with all OEMs). Moreover, participants were tested using a varying number of the vehicles. Consequently, a planned missing data design (e.g., Graham, Taylor, Olchowski, & Cumsille, 2006; Little & Rhemtulla, 2013) was used where some cells in the factorial were missing and the number of vehicles driven by a participant was used in all linear mixed effects models presented subsequently, in order to control for any impact of this latter factor.

On average, participants were tested on 5 vehicles, with a range of 1–24 vehicles (e.g., one participant was tested in 24 of the vehicles). Thus, the total number of participants in the study (120) is a product of the number of participants per vehicle (24) and the average number of vehicles in which participants were tested is 5. The number of vehicles driven by a participant was associated with the overall demand score (b = − 0.02, *t* = − 3.38, *p* = < .001). However, the effect size of the number of vehicles driven was relatively small, accounting for ~ 10% of the variability between participants. Though modest, we retained the number of vehicles driven by participants in all linear mixed effects models presented subsequently, in order to control for any impact of this variable.

## Results

A bootstrapping procedure was used to estimate the 95% confidence intervals (CI) around each point estimate in the analyses reported below. The bootstrapping procedure used random sampling with replacement to provide a nonparametric estimate of the sampling distribution. In our study, there were 24 participants tested in each vehicle. The bootstrapping procedure involved generating 10,000 bootstrapping samples, each of which was created by sampling with replacement N samples from the original “real” data. From each of the bootstrap samples, the mean was computed and the distribution of these means across the 10,000 samples was used to provide an estimate of the standard error around the observed point estimate. Prior to bootstrapping all scores were baseline corrected, minimizing the potential for violations of homogeneity of variance in resampling procedures (e.g., Davison, Hinkley, & Young, 2003). The baseline correction eliminated any effects of participant in the analyses reported subsequently.

The greater the spread of the CI, the greater the variability associated with the point estimate. The obtained 95% CI also provides a visual depiction of the statistical relationship between the point estimate and the single-task baseline and/or the high demand referents for cognitive, visual, subjective, and interaction time. For example, if the high demand referent does not fall within the 95% CI, then the point estimate significantly differs from that referent. Similarly, if the 95% CI of two condition do not overlap, then the two conditions differ significantly. However, the 95% CI of two conditions may overlap and the differences may still be significant. In this case, if the pair-wise difference between two conditions divided by the pooled standard error exceeds *t* (23) = 2.064, the difference is significant at the *p* < .05 level (two tailed).

The standardized scores for the high demand cognitive or visual referent tasks can also be translated into effect size estimates (i.e., Cohen’s *d*). For cognitive demand, a standardized score of 1.0 reflects a Cohen’s *d* of 1.423. For visual demand, a standardized score of 1.0 reflects a Cohen’s *d* of 1.519. The high demand estimates for cognitive and visual referent tasks reflect *very large* effect sizes. Note that a standardized score of 2 would reflect a doubling of the effect size estimates, a standardized score of 3 would reflect a tripling of the effect size estimates, and so on. Note also that the effect size estimates for the high cognitive and visual demand are virtually equivalent (differing by less than 0.1 Cohen’s *d* units).

Linear mixed effects analyses were performed using R 3.3.1 (R Core Team, 2016), lme4 (Bates, Maechler, Bolker, & Walker, 2015), and multcomp (Hothorn, Bretz, & Westfall, 2008). In the analyses reported below, task type, modality, task type by modality, and vehicle were entered independently. The number of vehicles driven by the participant was entered as a fixed effect while participant, vehicle, modality, and task type were entered as random effects. In each case, *p* values were obtained by likelihood ratio tests comparing the full linear mixed effects model to a partial linear mixed effects model without the effect in question. This linear mixed modeling analysis has the advantage of analyzing all available data while adjusting fixed effect, random effect, and likelihood ratio test estimates for missing data.

In the first section of the results, the data are collapsed over the participants and vehicles to provide an understanding of how workload varied as a function of the task type and mode of IVIS interaction. These analyses are important because they document the demand of the task types and modes of interaction on driver workload independent of vehicle. The last section presents data at the vehicle level.

### Effects of task type

Table 2 presents the workload associated with the four IVIS task types evaluated in the on-road testing. Table 2 presents the cognitive demand, Table 2 presents the visual demand, Table 2 presents the subjective demand, and Table 2 presents the task interaction time. The overall demand is presented in Fig. 6.

**Table 2:** Cognitive, visual, subjective, and temporal demand as a function of task type

Task Type	Mean	Lower	Upper
Cognitive demand of task types			
Audio entertainment	1.21	1.17	1.24
Calling and dialing	1.13	1.10	1.16
Text messaging	1.19	1.16	1.23
Navigation	1.16	1.11	1.21
Visual demand of task types			
Audio entertainment	1.22	1.18	1.25
Calling and dialing	1.04	1.01	1.08
Text messaging	1.02	0.98	1.07
Navigation	1.32	1.26	1.38
Subjective demand of task types			
Audio entertainment	0.81	0.78	0.84
Calling and dialing	0.78	0.75	0.81
Text messaging	0.83	0.79	0.87
Navigation	0.96	0.90	1.01
Temporal demand of task types			
audio entertainment	0.78	0.76	0.79
Calling and dialing	0.93	0.91	0.95
Text messaging	1.28	1.24	1.31
Navigation	1.84	1.79	1.89

Cognitive demand was derived using Eq. 1. Inspection of Table 2 shows that the cognitive demand from each task type was greater than the N-back task (i.e., in each case the cognitive demand exceeded 1.0). The relative ordering of task types placed calling and dialing and the navigation task types as slightly less cognitively demanding than the audio entertainment and texting task types. This conclusion was confirmed by a significant difference in the fit of linear mixed effects models with and without task type included (χ^2^(3) = 14.08, *p* = .01).

Visual demand was derived using Eq. 2. A comparison of linear mixed effects models with and without task type indicated that task type was a significant predictor of visual demand (χ^2^(3) = 63.52, *p* < .01). Table 2 shows that the visual demand was not significantly different from the SuRT task for calling and dialing and text messaging task types, but was significantly higher than the SuRT referent for the audio entertainment and navigation task types. The overlap in confidence intervals indicates that the audio entertainment and navigation task types did not significantly differ in visual demand.

Subjective demand was derived using Eq. 3. A comparison of linear mixed effects models with and without task type indicated that task type was a significant predictor of subjective demand (χ^2^(3) = 56.17, *p* < .01). Table 2 shows that the subjective demand of all of the task types was less than the average of high-demand referent tasks. The relative ordering of the task types placed calling and dialing below the audio entertainment, texting, and the navigation task types. However, the overlap in confidence intervals indicates that the task types did not significantly differ in subjective demand with the exception of the contrast between calling and dialing and navigation.

Task interaction time was derived using Eq. 4. A comparison of linear mixed effects models with and without task type indicated that task type was a significant predictor of interaction time (χ^2^(3) = 2977.36, *p* < .01). Table 2 shows that text messaging and navigation task types took significantly longer than the 24-s interaction referent. The audio entertainment task type took significantly less time than the calling and dialing task type, which took less time to perform than the text-messaging task type. The longest task interaction times were associated with navigation, which took an average of approximately 40 s to complete.

**Fig. 5:**
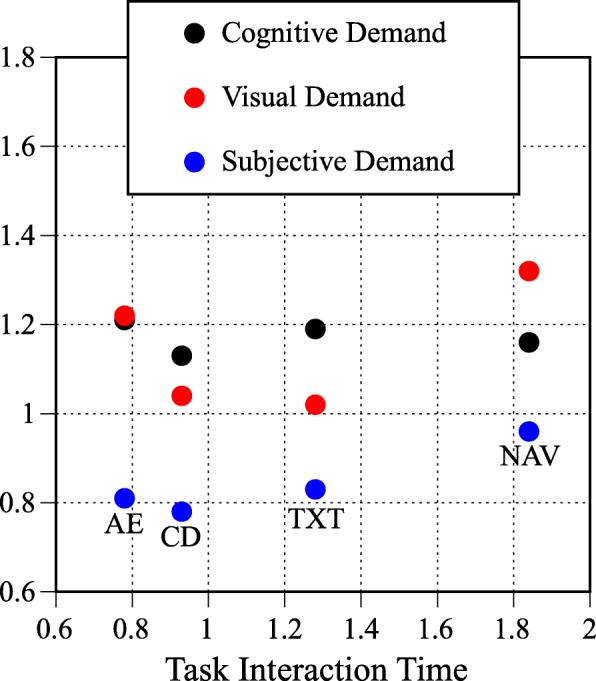
Mean task interaction time cross-plotted with cognitive, visual, and subjective demand for the four task types. AE, audio entertainment; CD, calling and dialing; TXT, text messaging; NAV, navigation. Note that the components comprising the overall demand rating are relatively independent and the different task types have different visual, cognitive, subjective, and temporal demand

Figure 5 cross-plots task interaction time with cognitive, visual, and subjective demand for the four task types. Note that the components comprising the overall demand rating are relatively independent and the different task types have different visual, cognitive, subjective, and temporal demand. For example, the visual and cognitive demand are nearly identical for the audio entertainment task type, cognitive demand is higher than visual demand for the calling and dialing and text messaging task types, and visual demand is higher than cognitive demand for the navigation task type. On the whole, subjective ratings are lower than the objective measures derived from the DRT.

**Fig. 6:**
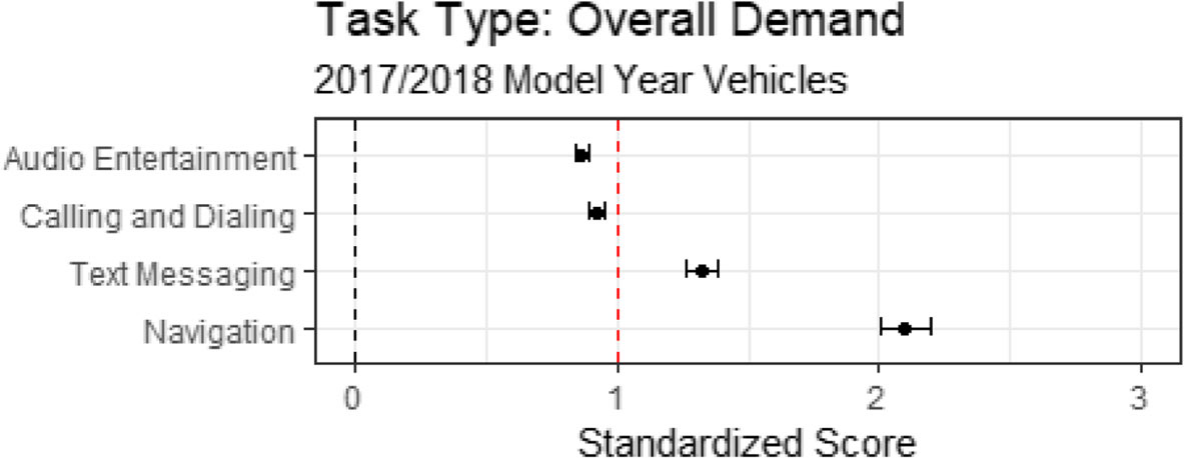
Overall demand as a function of task type for the on-road assessment. The dashed vertical black line represents single-task performance and the dashed vertical red line represents the high-demand referent tasks

Finally, overall demand, derived using Eq. 5 and presented in Fig. 6, shows that demand of the audio entertainment and calling and dialing task types fell below the high workload benchmark represented by the red vertical line and the text messaging and navigation task types exceeded the standardized high workload benchmark. A comparison of linear mixed effects models with and without task type indicated that task type was a significant predictor of overall demand (χ^2^(3) = 1244.65, *p* < .01). Of the four task types evaluated, audio entertainment and calling and dialing were the easiest to perform and they did not significantly differ in overall demand. Text messaging was significantly more demanding than audio entertainment and calling and dialing. The navigation task type was significantly more demanding than any of the other task types that were evaluated.

### Effects of modality of interaction

**Fig. 7:**
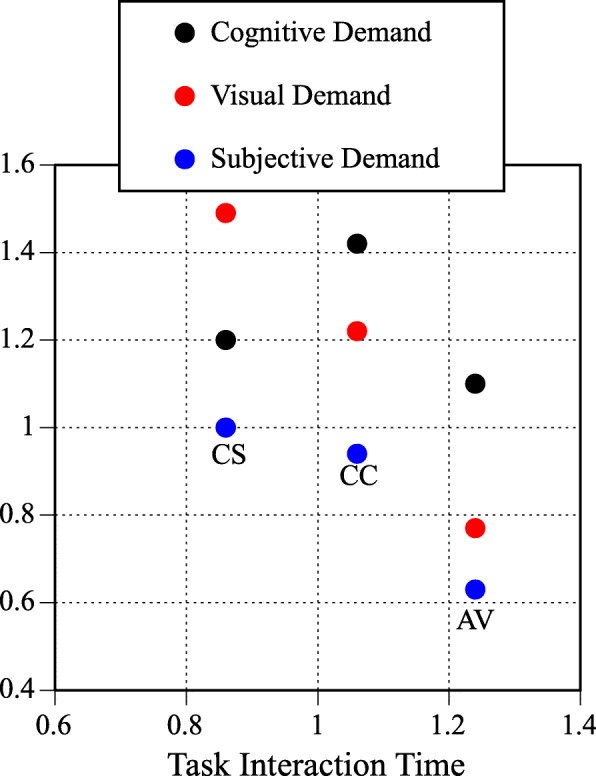
Mean task interaction time cross-plotted with cognitive, visual, and subjective demand for the three modalities. CS, center stack; CC, center console; AV, auditory vocal. Note that the components comprising the overall demand rating are relatively independent and the different modalities of interaction have different visual, cognitive, subjective, and temporal demand

**Fig. 8:**
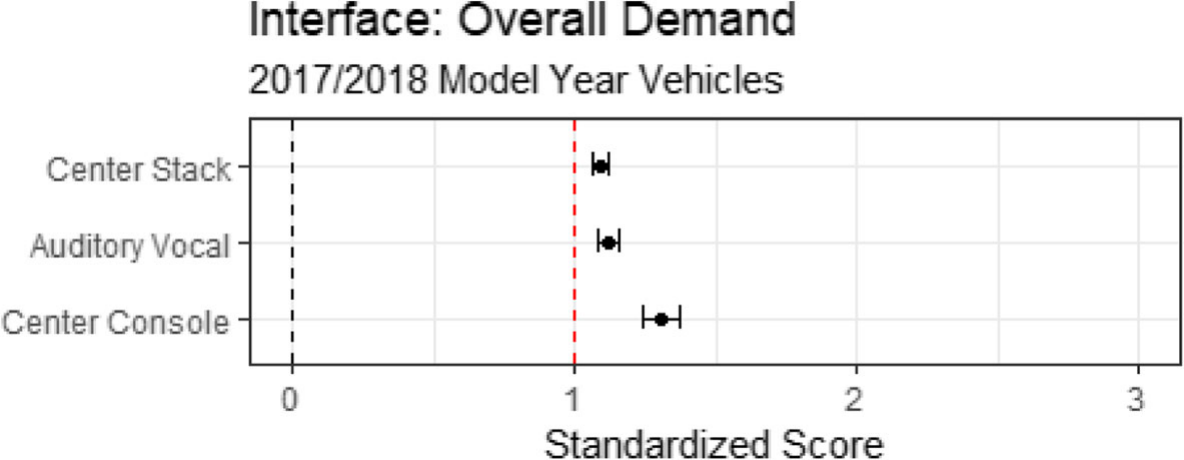
Overall demand as a function of modality for the on-road assessment. The dashed vertical black line represents single-task performance and the dashed vertical red line represents the high-demand referent tasks

Table 3 presents the workload associated with the three modes of interaction evaluated in the on-road testing. Table 3 presents the cognitive demand, visual demand, subjective demand, and task interaction time. Figure 7 cross-plots task interaction time with cognitive, visual, and subjective demand for the three modes of interaction and overall demand is presented in Fig. 8.

**Table 3:** Cognitive, visual, subjective, and temporal demand as a function of modality

Modality	Mean		Lower	Upper
Cognitive demand of modality				
Center stack	1.20		1.17	1.23
Auditory vocal	1.10		1.07	1.12
Center console	1.42		1.36	1.48
Visual demand of modality				
Center stack	1.49		1.46	1.52
Auditory vocal	0.77		0.75	0.80
Center console	1.22		1.16	1.29
Subjective demand of modality				
Center stack	1.00		0.97	1.02
Auditory vocal	0.63		0.61	0.66
Center console	0.94		0.89	1.00
Temporal demand of modality				
Center stack	0.86	0.84	0.84	0.88
Auditory vocal	1.24	1.22	1.22	1.27
Center console	1.06	1.03	1.03	1.09

Cognitive demand was derived using Eq. 1. A comparison of linear mixed effects models with and without modality indicated that modality was a significant predictor of cognitive demand (χ^2^(2) = 97.9, *p* < .01). Table 3 shows that the cognitive demand of each modality of interaction was greater than the N-back task. The relative ordering placed the auditory vocal interactions as less cognitively demanding than the center stack interactions, which were less demanding than the center console interactions.

Visual demand was derived using Eq. 2. A comparison of linear mixed effects models with and without modality indicated that modality was a significant predictor of visual demand (χ^2^(2) = 1380.05, *p* < .01). Table 3 shows that the visual demand was significantly lower than the SuRT task for the auditory vocal interactions, as expected, but was significantly higher than the SuRT task for the center console and center stack interactions. Center console interactions were less visually demanding than center stack interactions.

Subjective demand was derived using Eq. 3. A comparison of linear mixed effects models with and without modality indicated that modality was a significant predictor of subjective demand (χ^2^(2) = 548.15, *p* < .01). Table 3 shows that the subjective demand was lower than the high-demand referent tasks. Auditory vocal interactions were subjectively less demanding than center console and center stack interactions. Center console interactions were subjectively less demanding than center stack interactions.

The interaction time was derived using Eq. 4. A comparison of linear mixed effects models with and without modality indicated that modality was a significant predictor of interaction time (χ^2^(2) = 1063.48, *p* < .01). Table 3 shows that center stack interactions took significantly less time than the 24-s standard and auditory vocal tasks took significantly more time than the 24-s standard. Center console interaction time did not differ significantly from the 24-s standard.

Finally, overall demand, derived using Eq. 5 and presented in Fig. 8, shows that all the tasks exceeded the standardized high workload referent represented by the red vertical line. A comparison of linear mixed effects models with and without modality indicated that modality was a significant predictor of overall demand (χ^2^(2) = 18.75, *p* < .01). Of the three modes of interaction evaluated, center stack interactions were the easiest to perform. Auditory vocal interactions were more demanding than center stack interactions. The center console was the most demanding mode of interaction that we evaluated.

### Effects of vehicle

**Fig. 9:**
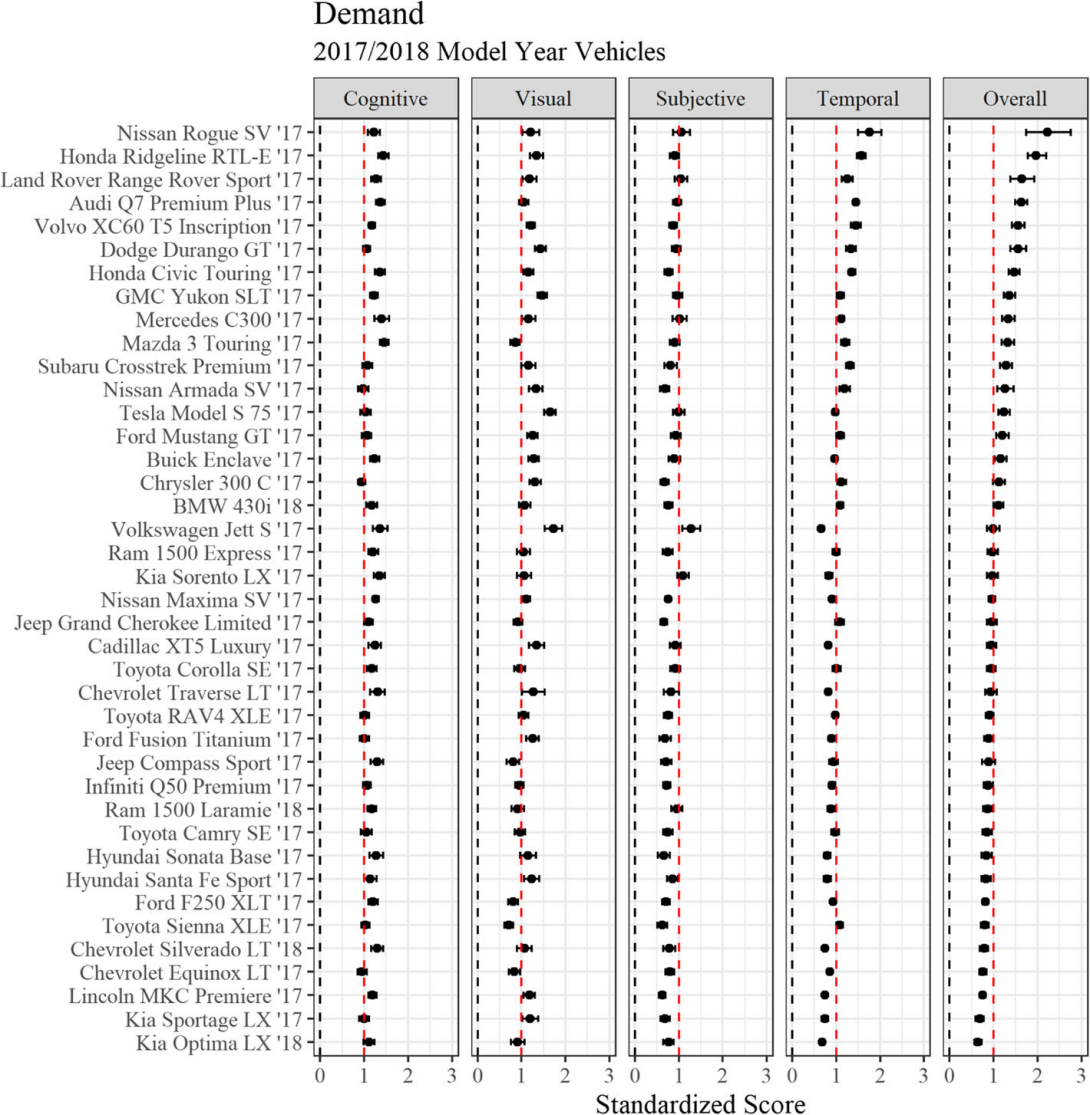
Demand as a function of vehicle for the on-road assessment. Vehicles are ordered by increasing levels of overall demand. The dashed vertical black line represents single-task performance and the dashed vertical red line represents the high demand referent. Error bars represent 95% confidence intervals

Figure 9 presents the workload associated with the different vehicles evaluated in the on-road testing. In the figure, vehicles are ordered by increasing levels of overall demand. Cognitive demand was derived using Eq. 1. A comparison of linear mixed effects models with and without vehicle indicated that vehicle was a significant predictor of cognitive demand (χ^2^(39) = 196.00, *p* < .01). Visual demand was derived using Eq. 2. A comparison of linear mixed effects models with and without vehicle indicated that vehicle was a significant predictor of visual demand (χ^2^(39) = 379.11, *p* < .01). Subjective demand was derived using Eq. 3. A comparison of linear mixed effects models with and without vehicle indicated that vehicle was a significant predictor of subjective demand (χ^2^(39) = 206.81, *p* < .01). Task interaction time was derived using Eq. 4. A comparison of linear mixed effects models with and without vehicle indicated that vehicle was a significant predictor of interaction time (χ^2^(39) = 1038.62, *p* < .01).

Overall demand, derived using Eq. 5, shows that the majority of vehicles were at or exceeded the standardized high workload benchmark represented by the red vertical line. A comparison of linear mixed effects models with and without vehicle indicated that vehicle was a significant predictor of overall demand (χ^2^(39) = 594.20, *p* < .01). There is a noticeable positive skew in the overall demand ratings. Twelve of the vehicles received an overall rating significantly below 1.0 (i.e., a moderate level of overall demand); 13 vehicles received a score that did not differ from the high-demand referent (i.e., a high overall demand score), and 15 vehicles scored significantly above the high-demand referent (i.e., an extreme overall demand score).

## Discussion

New automobiles provide an unprecedented number of features that allow motorists to perform a variety of secondary tasks unrelated to the primary task of operating a motor vehicle. Surprisingly, little is known about how these complex multimodal IVIS interactions impact the driver’s workload. Given the ubiquity of these systems, the current research used cutting-edge methods to address three interrelated questions concerning this knowledge gap.^2^

First, are some task types more impairing than others? The answer to this question can be seen most directly in Fig. 6, which plots overall demand as a function of task type. In that figure, the overall workload associated with audio entertainment and calling and dialing task types was lower than the high-demand referent, standardized as a score of 1.0 and indicated by a red vertical line. Text messaging and the navigation task types were more demanding than the high-demand referent. The task types differed in terms of demand, with audio entertainment task type being statistically equivalent to the calling and dialing task type (the two most universal of tasks available in all the automobiles we tested). Text messaging, a feature found in 26 out of 40 vehicles we tested, was associated with a significantly higher level of demand than the high demand referent. Most demanding of all was destination entry for navigation, a feature that was available in 14 out of 40 of the vehicles we evaluated. The navigation task type had an overall demand that was more than twice that of the high-demand referent.

One critical factor for the high workload ratings was the interaction time. The shortest interaction times were associated with audio entertainment. Calling and dialing took significantly longer than the selection of music. Texting took an average of 30 s, and destination entry for navigation took an average of 40 s. Clearly, the latter two task types divert the driver’s attention from the road for far too long. For example, at 25 mph, drivers would travel just under 1500 ft (over a quarter of a mile) while entering destinations for navigation and several of the systems that were tested took considerably longer than the 40-s average.

Of note were the subjective ratings, which tracked reasonably well with the measures of cognitive and visual demand, but not with interaction time. For example, the subjective demand rating for the navigation task did not differ from the audio entertainment task, despite a more than 2:1 difference in interaction time. Data such as these call into question assumptions that motorists are capable of self-regulating their secondary-task behavior (see Sanbonmatsu, Strayer, Biondi, Behrends, & Moore, 2016). That is, from the driver’s *subjective* perspective, the two tasks were very similar, whereas the measures of overall demand associated with *objective* measures tells a very different story.

Second, are some modes of interaction more distracting than others? The answer to this question can be seen in Fig. 8, which plots overall demand as a function of the mode of interaction. The overall workload associated with each mode of interaction was greater than the high-workload referent, standardized as a score of 1.0 and indicated by a red vertical line. Interactions using the center stack were significantly less demanding than auditory vocal interactions, which were less demanding than center console interactions. Interestingly, using voice-based commands to control IVIS functions resulted in significantly lower levels of visual demand than the SuRT task. By design, auditory-vocal interfaces allow the driver to keep their eyes on the road while interacting with the IVIS; however, with this type of interaction motorists are less likely to see what they are looking at (Strayer et al., 2003). Unfortunately, the benefits of reduced visual demand were offset by longer interaction times. Auditory vocal interactions took significantly longer than any other IVIS interaction (an average of 30 s in our testing).

Third, are IVIS interactions easier to perform in some vehicles than others? As illustrated in Fig. 9, there were surprisingly large differences between vehicles in the overall demand of IVIS interactions. Of the 40 vehicles, 12 received an overall rating significantly below 1.0 (i.e., a moderate level of overall demand). Of the 40 vehicles, 13 received a score that did not differ from the high-demand referent (i.e., a high overall demand score). Of the 40 vehicles, 15 scored significantly above the high-demand referent (i.e., a very high overall demand score). On the whole, vehicles in the latter category tended to have higher levels of demand on cognitive, visual, and subjective measures as well as longer interaction times.

The vast majority of the IVIS features we evaluated were unrelated to the task of driving (or, in the case of destination entry to support navigation, could have been performed *before* the vehicle was in motion). These IVIS interactions were often associated with high levels of cognitive and visual demand and long interaction times. Our objective assessment indicates that many of these features are just too distracting to be enabled while the vehicle is in motion. Greater consideration should be given to what IVIS features *should* be available to the driver when the vehicle is in motion rather than to what IVIS features *could* be available to motorists.

### Theoretical considerations

In the introduction, we outlined two theoretical accounts for why dual-task interference occurs (e.g., Bergen et al., 2014). On the one hand, domain-general accounts attribute dual-task interference to a competition for general computational or attentional resources that are distributed flexibly between the various tasks (e.g., Kahneman, 1973; Navon & Gopher, 1979). When two tasks require more resources than are available, performance on one or both of the tasks is impaired. On the other hand, domain-specific accounts attribute dual-task interference to competition for specific computational resources. The more two similar tasks are, in terms of specific processing resources, the greater the interference, or “code conflict” (e.g., Navon & Miller, 1987), or “crosstalk” (e.g., Pashler, 1994).

**Fig. 10:**
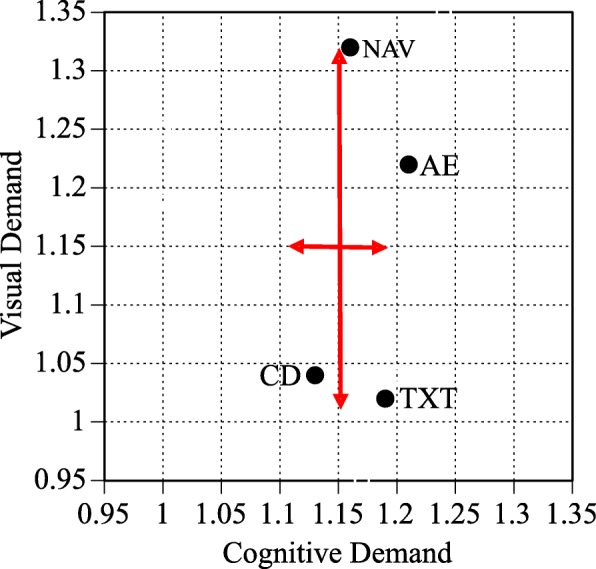
Cross-plot of the cognitive and visual demand for the four task types. AE, audio entertainment; CD, calling and dialing; TXT, text messaging; NAV, navigation. The horizontal and vertical red arrows represent the spread of cognitive and visual demand ratings for the four task types. Note the smaller range in cognitive demand than visual demand for the four task types

Figure 10 cross-plots the cognitive and visual demand for the four task types that were evaluated in the current research. The horizontal and vertical red arrows in the figure represent the variation in cognitive and visual demand, respectively, for the four task types. There is clearly a much smaller range in cognitive demand than visual demand for the four task types. Following Bergen et al. (2014), the consistency in the cognitive demand ratings across the four task types provides evidence for domain-general interference. Despite differences in visual demand and the time needed to perform an operation, cognitive demand was largely invariant, suggesting that performing any of these task types place a similar demand on a limited-capacity fungible resource. That is, relative to the single task of driving the vehicle, when participants were performing any one of these four task types, the cognitive demand was consistently high.

By contrast, there was much greater variability in visual demand for the four task types providing evidence for domain-specific interference. The calling and dialing and text-messaging task types placed significantly less demand on visual resources than the audio entertainment and navigation task types. This pattern cannot be chalked up to task difficulty per se, because this differential pattern was not observed with the cognitive demand (where the demand was largely invariant when participants performed the same four task types). Moreover, of the four task types, task completion time was shortest for audio entertainment and longest for navigation, yet these two had the highest visual demand. Thus, total task interaction time by itself was not a determining factor in the task differences. Furthermore, the pattern of data cannot be attributed to differential sensitivity of the cognitive and visual demand metrics, because the effect size for the high-demand referents tasks, N-back and SuRT, respectively, were equivalent in magnitude. The data indicate that the more complex visual search requirements of the audio entertainment and navigation task types made them more demanding than the calling and dialing and text messaging tasks. Given that visual demand was derived from miss rates of the DRT light projected on the windshield, some portion of the difference stems from a longer diversion of visual attention (and the eyes) from the forward roadway to perform the two more demanding tasks (Wickens, 2008).

### Limitations and caveats

The current research provided separate estimates of structural (i.e., visual demand) and attentional (i.e., cognitive demand) sources of interference. However, few, if any, tasks are process pure (Jacoby, 1991). Even the SuRT task used in the current study, while placing heavy demands on visual-manual resources (i.e., the eyes and hands), nevertheless placed minimal demands on limited capacity attention. Similarly, there is nothing for the driver to touch or see with the N-back task, yet it alters the visual scanning pattern of motorists (for a review, see Strayer & Fisher, 2016). Moreover, the dependent measures are not “pure” either. For example, while the hit rate of the visual DRT is sensitive to eyes off the road (and produces similar patterns to those obtained with eye tracking measures), the literature on inattention blindness shows that motorists can look directly at something and fail to “see” it (e.g., Strayer et al., 2003) because attention is diverted elsewhere.

Our research instructed participants to perform the IVIS tasks in an experimental order that was counterbalanced across participants and vehicles. This method provides an ability to make causal statements on different IVIS activities and the workload associated with them. However, in real-world settings, drivers are free to perform the IVIS tasks if, when, and where they so choose. This complicates the relationship between driver workload as measured in experimental studies and crash risk. For example, motorists may attempt to self-regulate their non-driving activities to periods where they perceive the risks to be lower. However, self-regulation depends upon drivers being aware of their performance and adjusting their behavior accordingly. This ability is often limited by the same factors that caused motorists to be distracted in the first place (e.g., see Sanbonmatsu et al., 2016).

We selected as high-demand referent tasks the N-back (2-back) and SuRT tasks, and adopted the 24-s rule for dynamic task interaction time. IVIS interactions (for tasks, modes of interaction, and vehicles with lower demand than these referent tasks scored well whereas those with higher demand than the referent tasks scored poorly. One may question whether the referents are reasonable. That is, if the referent tasks were too easy (or hard), then the *absolute* ratings would be an overestimate (or underestimate) of the true demand.^3^ Note that the *relative* ratings of tasks, modes of interaction, and vehicles should be insensitive to the absolute demand of the referent tasks, so long as they are performed in a consistent fashion in a counterbalanced order across participants.

The 24-s task interaction referent is derived from the NHTSA visual/manual guidelines (National Highway Traffic Safety Administration, 2013). Video coding of eye glances when participants performed the SuRT task and indicated that they took their eyes off the road 50% of the time when performing the SuRT task. Thus, performance of the high visual/manual demand SuRT for 24 s, a score of 1.0 in our rating system, matches the NHTSA acceptable limit. However, changes in the task interaction time referent will alter the *absolute* ratings; however, the *relative* rank ordering will not change.

Finally, the separation of structural and attentional interference may be useful for designers to help minimize distraction, so long as there is a realization that both facets of distraction are important to mitigate. Moving from simple button presses to voice commands without a careful analysis of the costs and benefits may have unintended consequences. For example, we found that using voice commands reduced visual demand, but at a cost of considerably longer interaction times. In many instances a 2-s button press is preferable to a 20 s voice-based interrogatory to perform the same task (see also Kidd, Dobres, Reagan, Mehler, & Reimer, 2017).

## Conclusions

The last decade has seen an extraordinary increase in the digital technology at the motorist’s fingertips that facilitate multimodal interactions that are unrelated to the task of driving. New vehicles are equipped with (at least) one LCD screen in the center stack that often supports touchscreen interactions with complex menus. All vehicles have some form of voice-command system that allows motorists to push a button and speak to initiate an interaction. Some vehicles include more distinctive configurations (e.g., write pads, rotary dials, gesture controls, heads-up displays, etc.). Given the ubiquity of these systems, the current research addressed three interrelated questions concerning this knowledge gap. First, are some tasks more impairing than others? Second, are some modes of interaction more distracting than others? Third, are IVIS interactions easier to perform in some vehicles than others? The answer to each question is yes. Tasks vary in visual, cognitive, and subjective demand and in the time to perform the actions. Interaction modalities also differ significantly in demand. Finally, vehicles differed considerably in the demand associated with IVIS interactions. Some of the demand stems from the tasks and modes of interaction supported by different OEMs. Other sources of demand were associated with awkward and confusing human-machine interfaces. Often, the time to perform an IVIS interaction was excessive. Many of the more complex IVIS features and functions were associated with extreme levels of overall demand.

We recommend that automakers consider which IVIS features *should* be available rather than *could* be available when the vehicle is in motion. For example, the NHTSA visual-manual guidelines (National Highway Traffic Safety Administration, 2013, p. 116) recommend against in-vehicle electronic systems that allow drivers to perform the following activities when the vehicle is moving:

- Visual-manual text messaging
- Visual-manual Internet browsing
- Visual-manual social media browsing
- Visual-manual navigation system destination entry by address
- Visual-manual 10-digit phone dialing
- Displaying more than 30 characters of text unrelated to the task of driving

Our testing found several instances in which drivers could perform the multimodal interactions listed above while the vehicle was in motion. Notably, vehicles that supported these features when the vehicle was in motion were often associated with the higher demand ratings. Locking out these activities when the vehicle is in motion and shortening the task interaction time are two methods that would reduce the overall demand of the IVIS interactions.

## Additional file

Additional file 1 Command syntax for the different tasks performed in each vehicle. (DOCX 73 kb)

## Footnotes

1 Here we refer to cognitive demand as the mental workload associated with performing IVIS interaction when the vehicle is in motion. This would include perception, attention, memory, decision-making, and response-related processes. By contrast, visual demand would include the structural interference associated with taking the eyes off the forward roadway as well as the central interference in visual processing that arises from IVIS interactions.

2 An earlier technical report based on two thirds of the vehicles that were evaluated in this paper (i.e., Strayer et al., 2017b) arrived at similar conclusions, further documenting the robustness of the findings reported in this manuscript.

3 The two high-demand referent tasks have a well-established record for creating high levels of cognitive demand (e.g., Mehler et al., 2011) and visual demand (e.g., Engström & Markkula, 2007; Mattes et al., 2007). In fact, the effect size estimates of the N-back and SuRT tasks were very large and of equivalent magnitude (i.e., Cohen’s *d* was 1.423 and 1.519, respectively).
