# Supplementary material for: Assessing the visual and cognitive demands of in-vehicle information systems
Source: Cogn Res Princ Implic. 2019 Jun 21;4:18. doi: 10.1186/s41235-019-0166-3 (PMC6588669; doi:10.1186/s41235-019-0166-3)
Supplement: Supplementary file 1 — Command syntax for the different tasks performed in each vehicle. (DOCX 73 kb) [file 41235_2019_166_MOESM1_ESM.docx]

**APPENDIX 1**

**A. Audio Entertainment Tasks *all***

- **Radio frequency tuning**
- **iPod contents (songs, artists, albums, genres)**

| 1. Choose a **Jazz song** from the iPad 2. Play **1020 AM** 3. Tune the radio to **98.5 FM** 4. Listen to the song **“99 Red Balloons”** 5. The **band “Nirvana”** is what you want to hear 6. Change the radio to your favorite **FM station** 7. Turn on the “**Metal” genre** 8. Let’s hear the **song “I’m Gonna Be (500 Miles)”** 9. You want to hear one of your favorite **AM stations** 10. Tune **AM 1540** 11. **89.1** 12. Tune to **1240** 13. iPod play **album “Storyline”** | 1. **AM 1160** 2. **Play a song by the artist “Eminem”** 3. Play the **album “Homesick”** 4. **90.1** 5. You want “**Johnny Cash” songs** to play 6. Radio **1630** 7. **“Riptide” is a song you want to play** from the iPod 8. Switch the iPod to **artist “Louis Armstrong”** 9. Play the “**Alternative” genre** 10. Change the **genre** to **“Reggae”** 11. Radio tune to **97.1 FM** 12. You want to hear a song by the **artist “Hunter Hayes”** 13. Change the music to the **song “Three Little Birds”** 14. Listen to **FM 99.5** |
| --- | --- |

**B. Audio Entertainment Tasks *Radio and iPod as source***

- **Radio frequency tuning**
- **iPod source (no content access)**

| 1. Choose a song from the **iPad** 2. Play **1020 AM** 3. Tune the radio to **98.5 FM** 4. Listen to the song on the **iPod** 5. Change the radio to your favorite **FM station** 6. Turn on the **iPod music** 7. Let’s hear a song on the **iPod** 8. You want to hear one of your favorite **AM stations** 9. Tune **AM 1540** 10. **89.1** 11. Tune to **1240** | 1. iPod play 2. **AM 1160** 3. Play a song via **iPod** 4. **90.1** 5. You want **iPod** songs to play 6. Radio **1630** 7. You want to play music from the **iPod** 8. Radio tune to **97.1 FM** 9. You want to hear a song by your favorite artist on the **iPod** 10. Listen to **FM 99.5** |
| --- | --- |

**C. Audio Entertainment Tasks *Radio and iPod songs***

- **Radio frequency tuning**
- **iPod contents (songs only)**
- **Task is complete once media has been loaded.**

| 1. Choose a **song** from the iPad 2. Play **1020 AM** 3. Tune the radio to **98.5 FM** 4. Listen to the song **“99 Red Balloons”** 5. The **song “Not Afraid”** is what you want to hear 6. Change the radio to your favorite **FM station** 7. Turn on **“Mess Around” the song** 8. Let’s hear the **song “I’m Gonna Be (500 Miles)”** 9. You want to hear one of your favorite **AM stations** 10. Tune **AM 1540** 11. **89.1** 12. Tune to **1240** 13. iPod play **song “Storyline”** | 1. **AM 1160** 2. Play the **song “Don’t Stop Believin”** 3. Play the **song “I Can See Clearly Now”** 4. **90.1** 5. You want **“If It Means Alot To You” song** to play 6. Radio **1630** 7. **“Riptide” is a song you want to play** from the iPod 8. Switch the iPod to **song “Let it Be”** 9. Play the **“Come Together”** 10. Radio tune to **97.1 FM** 11. Change the music to the **song “Three Little Birds”** 12. Listen to **FM 99.5** |
| --- | --- |

**D. Audio Entertainment Tasks *iPod only***

- **iPod contents (songs only)**
- **Task is complete once media has been loaded.**

| 1. Listen to a **Jazz song** 2. Play **the artist A Day to Remember** 3. Play your **Favorite Song** 4. Listen to the song **“99 Red Balloons”** 5. The **band “Nirvana”** is what you want to hear 6. Play a **Pop** music internet radio station 7. Turn on the “**Metal” genre** 8. Let’s hear the **song “I’m Gonna Be (500 Miles)”** 9. You want to hear one of your favorite **Artists** 10. You want to hear a song by **Adele** 11. **Somebody Else** 12. Tune to **a Country** internet radio station 13. Play the **album “Safe House”** | 1. **Pantera** 2. Play a **song** by the **artist “Eminem”** 3. Play the **album “Homesick”** 4. You want to hear the song “**I Can See Clearly Now”** 5. You want a “**Johnny Cash” song** to play 6. **Hip-Hop** Radio 7. **“Riptide” is a song** you want to play 8. Switch audio to **artist “Louis Armstrong”** 9. Play the “**Alternative” genre** 10. Change the **genre** to **“Reggae”** 11. **Wagon Wheel** 12. You want to hear a song by the **artist “Hunter Hayes”** 13. Change the music to the **song “Three Little Birds”** 14. Listen to **Justin Bieber** |
| --- | --- |

**E. Calling & Dialing Tasks *Contacts and Dialing***

- **Participant calls contacts (cell phone, work).**
- **Dials numbers (participant’s own phone number^+^, 801-555-1234).**
- **Task is complete once call has been successfully ended.**

| 1. **Jack Olsen** would like you to call him on his **cell phone** 2. You need to call **8“OH”1-555-1234** 3. **Willow Brooks** 4. Try to reach **Brad Peterson** 5. Enter **8“ZERO”1-555-1234** 6. You can’t find **your phone**. Call it to find it. 7. Ring **Felicity Gomez’s office** 8. Enter your **own number** 9. You missed a call from **Oliver Reed** 10. Telephone **8“OH”1-555-1234** 11. **Violet Wheeler** is waiting to hear back from you on her **mobile** | 1. Dial your **own number** 2. Give **Phil Potter** a call back at work 3. Give **8“ZERO”1-555-1234** a call 4. Try **Helen Harold** on her **business number** 5. Call your **own phone** 6. **8“OH”1-555-1234** 7. **Bethany Swan, cell phone** 8. Telephone **Jennifer Long** 9. You need to talk to **Yolanda Chavez** 10. Dial **Tanya Henry** 11. Call **Andrew Fink’s mobile** back |
| --- | --- |

+RA asked for consent from participant to use participant’s phone number.

**F. Calling & Dialing Tasks *Contacts only***

- **Participant calls contacts (cell phone, work).**
- **Task is complete once call has been successfully ended.**

| 1. **Jack Olsen** would like you to call him on his **cell phone** 2. **Willow Brooks** 3. Try to reach **Brad Peterson** 4. Ring **Felicity Gomez’s office** 5. You missed a call from **Oliver Reed** 6. **Violet Wheeler** is waiting to hear back from you on her **mobile** 7. Give **Phil Potter** a call back at **work** 8. Try **Helen Harold** on her **business number** 9. **Bethany Swan, cell phone** 10. Telephone **Jennifer Long** 11. You need to talk to **Yolanda Chavez** | 1. Dial **Tanya Henry** 2. Call **Andrew Fink’s mobile** back 3. You need to call **Ian Gavin** 4. Place a call to **Frank Waterfall’s office** 5. You can’t reach **Francis Baker**. Call them again. 6. You need to reach **Eve Remington** 7. Telephone **Daniel Granger** 8. Dial **Alan Fink mobile** 9. Give **Mia Aston** a call 10. Call **Nathan Chow** again 11. **Oakley James** is waiting for your call |
| --- | --- |

**G. SMS *Read Only* Tasks**

- **Participant has system read out text messages.**
- **Task complete once message has been selected, not once message is done being read aloud.**

| 1. Read out the message from **Cam Whitman** 2. Read out the text from **Andy Cameron** 3. What did **Amelia Kidder** send you? 4. **Maggie Carter** just messaged you. 5. What did **Rachel Gatsby** say? 6. Find a message from **Andy Cameron** 7. **Scarlett Miles** sent you a new text 8. Read the text from **Amelia Kidder** 9. What did **Maggie Carter** send you? | 1. Read the text from **Cam Whitman** 2. New message from **Lucas Forester** 3. What did **Cam Whitman** send you? 4. Read out the message from **Maggie Carter** 5. What did **Scarlett Miles** send you? 6. What does the text from **Maggie Carter** say? 7. What did **Rachel Gatsby** send you? 8. What does the message from **Scarlett Miles** say? 9. New message from **Andy Cameron** |
| --- | --- |

**H. SMS *Read & Send* Tasks**

- **Participant reads and responds to texts with system-specific predetermined messages.**
- **Task is complete once message has been sent.**

| 1. Read out the message from **Cam Whitman**. Please respond. 2. Read and reply to the text from **Andy Cameron** 3. What did **Amelia Kidder** send you? Send your answer. 4. **Maggie Carter** just messaged you. What should you send back? 5. What did **Rachel Gatsby** say? Reply to her. 6. Find a message from **Andy Cameron**. Reply. 7. **Scarlett Miles** sent you a new text. Send something back. 8. Read the text from **Amelia Kidder** and respond to it. 9. What did **Maggie Carter** send you? Send a text back. | 1. Read and respond to the text from **Cam Whitman** 2. New message from **Lucas Forester**. How do you reply? 3. What did **Cam Whitman** send you? Answer him. 4. Read out the message from **Maggie Carter.** Send your reply. 5. What did **Scarlett Miles** send you? Text her back. 6. How do you respond to the text from **Maggie Carter**? 7. You need to read and reply to **Rachel Gatsby’s** message. 8. What does the message from **Scarlett Miles** say? Respond. 9. Read and then reply to the new message from **Andy Cameron.** |
| --- | --- |

**I. SMS *Send Only* Tasks**

- **Participant sends a new text message in response to the given scenario to a phone contact.**
- **Task is complete once message has been sent.**

| 1. Let **Hugo Grant’s Office** know you’re going to be late. 2. **Hunter Bowman** is asking if you want to go to the movies tonight. 3. **Eve Remington** wants to go dancing tonight. 4. **Milly Jung** texted you a funny joke. 5. Text **Kevin Malcome** to ask for directions. 6. Ask **Quinn Brown (Cell)** where they are. 7. Tell **Paige Green** you’re too busy driving to text right now. 8. Tell **Landon Carter** to text you. 9. **Vince Hancock** texted you a silly dad joke. 10. **Brad Peterson** has big news and is wondering if you can talk right now. | 1. **Zoe Ferris** dropped off your favorite cookies at your house. 2. **Isabelle Morales** is wondering where you are. 3. **Natalie Ling** can pick you up from the airport next week. 4. Tell **Jack Olsen** to call you from **work** 5. Tell **Willow Brooks** you’re too busy driving to call them right now. 6. **Francis Baker** wants to know if they can copy your homework. 7. **Milo Santiago** wants to know why you’re not at the restaurant yet. 8. **Gretchen Warner** says they will clean your car for you tonight. |
| --- | --- |

**J. Navigation Tasks**

- **Participant sets the destination to a point of interest that best fits the task goal.**
- **Participant cancels the route before the task is considered to be complete.**

| 1. **(Gas)** Fill up at the closest gas station. 2. **(Library)** Your library book is overdue. Let’s return it at the closest library. 3. **(Italian Restaurant)** You’re headed out for some Italian food at nearby restaurant. 4. **(Coffee shop)** Grab yourself a cup of coffee from the closest Starbucks. 5. **(Grocery store)** You need some items from Whole Foods. 6. **(ATM\bank)** You need to get cash from a Wells Fargo bank. 7. **(Mexican Restaurant)** Find a Mexican restaurant nearest you. | 1. **(Hospital)** Go visit your friend at the LDS Hospital. 2. **(Chinese Restaurant)** You’re craving food from Panda Express. 3. **(Movie theater)** You’re on your way to see a movie at the nearby theater. 4. **(Hotel/Motel)** Drive to the nearest lodging to stay the night. 5. **(Post office)** You have a package to drop off at the closest Post Office. 6. **(Museum)** Go check out the new exhibit at the Utah Museum of Natural History. 7. **(Shopping Center)** Go pick out some new clothes at a nearby shopping mall. |
| --- | --- |

**K. Navigation Tasks (with other locations)^+^**

- **Participant sets the destination to a point of interest that best fits the task goal.**
- **Participant cancels the route before the task is considered to be complete.**

| 1. **(Gas)** Fill up at the closest gas station. 2. **(Bowling)** It’s league night at the bowling alley. Don’t be late! 3. **(Restaurant)** You’re headed out for some pizza at nearby restaurant. 4. **(Coffee shop)** Grab yourself a cup of coffee from the closest cafe. 5. **(Car wash)** Go treat your car to a nice wash and wax. 6. **(ATM\Bank)** You need to get cash from a Wells Fargo bank. 7. **(Restaurant)** Enjoy a bagel from Einstein’s. | 1. **(Hospital)** Go visit your friend at the hospital. 2. **(Golf)** Play 18 holes at a Bonneville Golf Course. 3. **(Bar)** You’re meeting up with your friends at a nearby bar. 4. **(Hotel/Motel)** Drive to the nearest lodging to stay the night. 5. **(Police station)** You have to pay a parking ticket at the police station. 6. **(Museum)** Check out the new rides at Seven Peaks Waterpark. 7. **(Rest area)** Take a break at Jordanelle Rest Area. |
| --- | --- |

^+^Use these destinations if vehicle does not support destinations from list J.

**L. Navigation Tasks (with other locations)^+^**

- **Participant sets the destination to a point of interest that best fits the task goal.**
- **Participant cancels the route before the task is considered to be complete.**

| 1. **(Gas)** Fill up at the closest gas station. 2. **(Bookstore)** You are eager to buy a new book. Let's find a bookstore. 3. **(Breakfast Restaurant)** You’re headed out for some breakfast at a nearby restaurant. 4. **(Coffee shop)** Grab yourself a cup of coffee from the closest Starbucks. 5. **(Pharmacy)** You need some items from the local pharmacy. 6. **(Camping/ RV parks)** You need to be in nature, find the closest camping spot. | 1. **(Car wash)** Go treat your car to a nice wash and wax. 2. **(Bar)** You’re meeting up with your friends at the cocktail lounge, Bourbon House. 3. **(Airport)** You’re on your way to pick up a friend at the Salt Lake International Airport. 4. **(Hotel/Motel)** Drive to the nearest lodging to stay the night. 5. **(Shopping Center/Mall)** Go on a shopping date at Trolley Square. |
| --- | --- |

^+^Use these destinations of vehicle does not support destinations from list J or K.

**M. Audio Entertainment Tasks *Radio and iPod contents***

- **Radio frequency tuning**
- **iPod contents (songs, artists, albums, genres)**

| 1. Tune the radio to 90.1 FM 2. Tune the radio to 1230 AM 3. Play **Michael Jackson “Thriller”** 4. Tune the radio to 94.1 FM 5. Tune the radio to 530 AM 6. Play a **Pop genre** song 7. Tune the radio to 98.1 FM 8. Tune the radio to 1160 AM 9. Play **The Beatles “Let It Be”** 10. Tune the radio to 830 AM 11. Tune the radio to 96.3 FM 12. Play an **Alternative genre** song | 1. Tune the radio to 1320 AM 2. Tune the radio to 107.9 FM 3. Play **Katy Perry “Rise”** 4. Tune the radio to 1490 AM 5. Tune the radio to 103.5 6. Play a **‘Country’ genre** song 7. Tune the radio to 820 AM 8. Tune the radio to 96.7 FM 9. Play **Adele “Send My Love”** 10. Tune the radio to 1550 AM 11. Tune the radio to 98.1 FM 12. Play a **Rock genre** song |
| --- | --- |

**N. Audio Entertainment Tasks *Radio and iPod SONGS***

- **Radio frequency tuning**
- **iPod songs only**

| 1. Tune the radio to 90.1 FM 2. Play the song **‘Thriller’** 3. Tune the radio to 530 AM 4. Play the song **‘Let it Be’** 5. Tune the radio to 97.1 FM 6. Play the song **‘Come Together’** 7. Tune the radio to 1620 AM 8. Tune the radio to 96.3 FM 9. Play the song **‘Don’t Stop Believing’** 10. Play the song **‘The Funeral’** | 1. Tune the radio to 107.9 FM 2. Play the song **‘Rise’** 3. Tune the radio to 820 AM 4. Play the song **‘Billie Jean’** 5. Tune the radio to 89.1 FM 6. Tune the radio to 1620 AM 7. Play the song **‘Send my Love’** 8. Tune the radio to 98.1 FM 9. Play the song **‘H.O.L.Y.’** 10. Tune the radio to 610 AM |
| --- | --- |

**O_1_. Audio Entertainment Tasks *Radio, iPod contents and Bluetooth***

- **Radio frequency tuning**
- **iPod contents (songs, artists, albums, genres)**
- **Bluetooth audio source**

| 1. Tune to 90.1 FM 2. Change source to Bluetooth Audio 3. Play **Michael Jackson’s ‘Thriller’** 4. Tune to 94.1 FM 5. Tune to 530 AM 6. Change source to **Bluetooth Audio** 7. Tune to 98.1 FM 8. Tune to 1160 AM 9. Play a **‘Pop’ genre** song 10. Tune to 830 AM 11. Tune to 96.3 FM 12. Play an **‘Alternative’ genre** song | 1. Tune to 1610 AM 2. Tune to 107.9 FM 3. Change source to **Bluetooth Audio** 4. Tune to 1490 AM 5. Tune to 103.5 FM 6. Play a **‘Country’ genre** song 7. Tune to 820 AM 8. Tune to 96.7 FM 9. Play **Adele’s ‘Send My Love’** 10. Tune to 1550 AM 11. Tune to 98.3 FM 12. Change source to **Bluetooth Audio** |
| --- | --- |

**O_2_. Audio Entertainment Tasks *Radio, iPod contents and Bluetooth***

- **Radio frequency tuning to presets only**
- **iPod contents (songs, artists, albums, genres)**
- **Bluetooth audio source**

| 1. Tune the radio to 90.1 FM 2. Tune the radio to 1320 AM 3. Play **Michael Jackson “Thriller”** 4. Tune the radio to 97.1 FM 5. Tune the radio to 530 AM 6. Play music via **Bluetooth** 7. Tune the radio to 98.1 FM 8. Tune the radio to 1160 AM 9. Play a **‘Pop’ genre** song 10. Tune the radio to 820 AM 11. Tune the radio to 96.3 FM 12. Play an **‘Alternative’ genre** song | 1. Tune the radio to 1620 AM 2. Tune the radio to 107.9 FM 3. Play music via **Bluetooth** 4. Tune the radio to 610 AM 5. Tune the radio to 99.5 FM 6. Play a **‘Country’ genre** song 7. Tune the radio to 820 AM 8. Tune the radio to 96.3 FM 9. Play **Adele’s ‘Send My Love’** 10. Tune the radio to 1160 AM 11. Tune the radio to 98.1 FM 12. Play music via **Bluetooth** |
| --- | --- |

**P. Audio Entertainment Tasks *Radio Only***

- **Radio frequency tuning**
- **Radio categories**

| 1. Tune the radio to 90.1 FM 2. Tune the radio to 1230 AM 3. Change Music Type to **Nostalgia** 4. Tune the radio to 94.1 FM 5. Tune the radio to 530 AM 6. Tune the radio to 97.1 FM 7. Change Music Type to **R&B** 8. Tune the radio to 1160 AM 9. Tune the radio to 101.9 FM 10. Change Music Type to **College** 11. Tune the radio to 830 AM 12. Tune the radio to 96.3 FM | 1. Tune the radio to 1320 AM 2. Tune the radio to 107.9 FM 3. Change Music Type to **Weather** 4. Tune the radio to 1490 AM 5. Change Music Type to **Adult Hits** 6. Tune the radio to 103.5 7. Tune the radio to 820 AM 8. Tune the radio to 96.7 FM 9. Change Music Type to **Country** 10. Tune the radio to 1550 AM 11. Tune the radio to 98.1 FM 12. Tune the radio to 610 AM |
| --- | --- |

**Q. Audio Entertainment Tasks *Radio and Bluetooth Audio***

- **Radio frequency tuning**
- **Radio categories**

| 1. Tune the radio to 90.1 FM 2. Tune the radio to 1230 AM 3. Change radio to **Satellite** 4. Tune the radio to 94.1 FM 5. Tune the radio to 530 AM 6. Tune the radio to 97.1 FM 7. Change radio to **Bluetooth Audio** 8. Tune the radio to 1160 AM 9. Tune the radio to 101.9 FM 10. Change radio to **Satellite** 11. Tune the radio to 830 AM 12. Tune the radio to 96.3 FM | 1. Tune the radio to 1320 AM 2. Tune the radio to 107.9 FM 3. Change radio to **Bluetooth Audio** 4. Tune the radio to 1490 AM 5. Change radio to **Satellite** 6. Tune the radio to 103.5 7. Tune the radio to 820 AM 8. Tune the radio to 96.7 FM 9. Change radio to **Bluetooth Audio** 10. Tune the radio to 1550 AM 11. Tune the radio to 98.1 FM 12. Tune the radio to 610 AM |
| --- | --- |

**R. Calling & Dialing Tasks *Contacts and Dialing***

- **Participant calls contacts (cell phone, work).**
- **Dials numbers (participant’s own phone number^+^, 801-555-1234).**
- **Task is complete once call has been successfully ended.**

| 1. Call **George Hudson** at **Work** 2. Dial your **own phone #** 3. Call **Matt Plumb’s** **Mobile** 4. Dial **801-555-1234** 5. Call **David Jones** at **Work** 6. Dial your **own phone #** 7. Call **Zane Thompson** 8. Dial **801-555-1234** 9. Call **Randall Jenkins’** **Mobile** 10. Call **William Dunn** at **Work** | 1. Dial your **own phone #** 2. Call **Wendy Darling** 3. Call **Jessica Day** 4. Dial **801-555-1234** 5. Call **Ethan Hawke** 6. Dial your **own phone #** 7. Call **Lisa Hamilton** 8. Dial **801-555-1234** 9. Call **Brittany Sanders’ Mobile** 10. Dial your **own phone #** |
| --- | --- |

+RA asked for consent from participant to use participant’s phone number.

**S. Calling & Dialing Tasks *Contacts only***

- **Participant calls contacts (cell phone, work).**
- **Task is complete once call has been successfully ended.**

| 1. Call **William Dunn** at **Work** 2. Call **Phil Dunphee** 3. Call **Jessica Day** 4. Call **Matt Plumb’s Mobile** 5. Call **Ethan Hawke** 6. Call **Brittany Sanders’ Mobile** 7. Call **Lisa Hamilton** 8. Call **Wendy Darling** 9. Call **George Hudson** at **Work** 10. Call **David Jones’ Mobile** | 1. Call **Anna Pearl** 2. Call **Randall Jenkins** at **Work** 3. Call **Ethan Hawke** 4. Call **Brittany Sanders** at **Work** 5. Call **David Jones’ Mobile** 6. Call **Phil Dunphee** 7. Call **Zane Thompson** 8. Call **Randall Jenkin’s Mobile** 9. Call **Anna Pearl** 10. Call **William Dunn** at **Work** |
| --- | --- |

**T. Calling & Dialing Tasks *Favorite Contacts***

- **Participant calls contacts that have been stored as favorites if system does not allow access to the phonebook while driving..**
- **Task is complete once call has been successfully ended.**

| 1. Call **William Dunn** 2. Call **Lisa Hamilton** 3. Call **David Jones** 4. Call **Zane Thompson** 5. Call **Randall Jenkins** 6. Call **Matt Plumb** 7. Call **Jessica Day** 8. Call **Wendy Darling** 9. Call **Zane Thompson** 10. Call **Ethan Hawke** | 1. Call **George Hudson** 2. Call **Lisa Hamilton** 3. Call **David Jones** 4. Call **Zane Thompson** 5. Call **Randall Jenkins** 6. Call **Matt Plumb** 7. Call **Jessica Day** 8. Call **Wendy Darling** 9. Call **Zane Thompson** 10. Call **Ethan Hawke** |
| --- | --- |

**U. SMS *Send Only* Tasks**

- **Participant sends a new text message. System only replies to most recent message in the inbox.**
- **Task is complete once message has been sent.**

| 1. Send “**Yes**” 2. Send “**Where are you?**” 3. Send “**I’m stuck in traffic**” 4. Send “**No**” 5. Send “**Can’t talk right now, I’m driving**” 6. Send “**Be there in 10 minutes**” 7. Send “**Can’t wait to see you**” 8. Send “**Too funny**” 9. Send “**Thanks**” 10. Send “**Call me. I’m driving.**” | 1. Send “**Be there in 20 minutes**” 2. Send “**Why?**” 3. Send “**I love you**” 4. Send “**Call you later. I’m driving.**” 5. Send “**I need more directions. Can you call me?**” 6. Send “**Too funny.**” 7. Send “**Call me. I’m driving.**” 8. Send “**Yes**” 9. Send “**No**” 10. Send “**I’m stuck in traffic.**” |
| --- | --- |

**V. SMS *Send New Message* Tasks**

- **Participant sends a new text message to a phone contact.**
- **Task is complete once message has been sent.**

| 1. Send “**Text me the address**” to **Jessica Day** 2. Send “**I’ll arrive soon**” to **Phil Dunphee** 3. Send “**Stuck in traffic**” to **David Jones’ Cell** 4. Send “**LOL**” to **Valentine Wiggin** 5. Send “**Where are you?**” to **Wendy Darling** 6. Send “**Thanks**” to **William Dunn** at **Work** 7. Send “**Yes**” to **Brittany Sanders’ Cell** | 1. Send “**No**” to **Ethan Hawke** 2. Send “**On my way**” to **Randall Jenkins** at **Work** 3. Send “**Call me**” to **Jessica Day** 4. Send “**LOL**” **David Jones** at **Work** 5. Send “**When?**” to **Wendy Darling** 6. Send “**Yes**” to **Anna Pearl** 7. Send “**Where?**” to **George Hudson’s Mobile** 8. Send “**Text me the address**” to **Jessica Day** |
| --- | --- |

**W. SMS *Read Only* Tasks**

- **Participant has system read out text messages.**
- **Task complete once message has been selected, not once message is done being read aloud.**

| 1. Read text from **Maddie McCarty** 2. Read text from **Sydney Mills** 3. Read text from **Camille Wheatley** 4. Read text from **Kelly Mckenzie** 5. Read text from **Andrea Campos** 6. Read text from **Rachelle Gatsby** 7. Read text from **Camille Wheatley** 8. Read text from **Sydney Mills** 9. Read text from **Andrea Campos** 10. Read text from **Rachelle Gatsby** | 1. Read text from **Andrea Campos** 2. Read text from **Kelly Mckenzie** 3. Read text from **Maddie McCarty** 4. Read text from **Camille Wheatley** 5. Read text from **Rachelle Gatsby** 6. Read text from **Sydney Mills** 7. Read text from **Maddie McCarty** 8. Read text from **Andrea Campos** 9. Read text from **Camille Wheatley** 10. Read text from **Kelly Mckenzie** |
| --- | --- |

**X_1_. Navigation Tasks**

- **Participant sets the destination to a point of interest that best fits the task goal.**
- **Participant cancels the route before the task is considered to be complete.**

| 1. Find the nearest **Dunkin’ Donuts** 2. Find the nearest **Mexican** 3. Find the nearest **Hospital** 4. Find the nearest **Chase Bank** 5. Find the nearest **Starbucks** 6. Find the nearest **Gas Station** 7. Find the nearest **Hotel** 8. Find the nearest **Coffee Shop** 9. Find the nearest **Wells Fargo** 10. Find the nearest **Library** | 1. Find the nearest **Gas Station** 2. Find the nearest **Museum** 3. Find the nearest **Post Office** 4. Find the nearest **Hilton Hotel** 5. Find the nearest **Breakfast** 6. Find the nearest **Barbecue** 7. Find the nearest **Whole Foods** 8. Find the nearest **Japanese** 9. Find the nearest **Coffee Shop** 10. Find the nearest **Best Western** |
| --- | --- |

**X_2_. Navigation Tasks**

- **Participant sets the destination to a point of interest that best fits the task goal.**
- **Participant cancels the route before the task is considered to be complete.**

| 1. Navigate to the closest **hospital** 2. Navigate to the closest **restaurant** 3. Navigate to the closest **hotel and motel** 4. Navigate to the closest **gas station** 5. Navigate to the closest **shopping center** 6. Navigate to the closest **ATM** 7. Navigate to the closest **theater** 8. Navigate to the closest **museum** 9. Navigate to the closest **coffee shop** 10. Navigate to the closest **police station** | 1. Navigate to the closest **city center** 2. Navigate to the closest **ski resort** 3. Navigate to the closest **golf course** 4. Navigate to the closest **embassy** 5. Navigate to the closest **campground** 6. Navigate to the closest **rest area** 7. Navigate to the closest **business facility** 8. Navigate to the closest **train station** 9. Navigate to the closest **tourist attraction** 10. Navigate to the closest **pharmacy** |
| --- | --- |

Use these destinations of vehicle does not support destinations from list X_1_.

**Y. Audi Q7 3.0 T Quattro Premium Plus Audio Entertainment Tasks**

- **Use the Draw Pad to select music**
- **Use the Alphabet Ribbon to search for music**
- **Use the Rotary Wheel to search for music**

| 1. Using the **Draw Pad**, tune the radio to 97.1 FM 2. Play the song “Send My Love To My New Lover” using the **Draw Pad** 3. Play the artist The Beatles using the **Alphabet Ribbon** 4. Using the **Frequency List and Presets**, tune the radio to 1160 AM 5. Play “25” using the **Album menu** 6. Play music via Bluetooth 7. Using the **Alphabet Ribbon**, tune the radio to FM 100.3 FM 8. Using the **Draw Pad**, play an Alternative genre song 9. Play the artist Katy Perry using the **Artist menu** 10. Using the **Frequency List and Presets**, tune the radio 1240 AM | 1. Play the song “Let It Be” using the **Alphabet Ribbon** 2. Play the artist “Justin Bieber” using the **Draw Pad** 3. Play music via Bluetooth 4. Using the **Frequency List and Presets**, tune the radio to 96.3 FM 5. Play the song Don’t Stop Believing using the **Alphabet Ribbon** 6. Using the **Draw Pad,** tune the radio to AM 1490 7. Play the artist Michael Jackson using the **Artist menu** 8. Play a ‘Country’ genre song using the **Genre menu** |
| --- | --- |

**Z. Audi Q7 3.0 T Quattro Premium Plus Calling & Dialing Tasks**

- **Participant dials numbers using:**
  - **Alphabet Ribbon**
  - **Draw Pad**
- **Participant calls contacts (work or cell phone)using:**
  - **Alphabet Ribbon**
  - **Draw Pad**
  - **Rotary Wheel**
- **Task is complete once call has been successfully ended.**

| 1. **Directory** Call John Smith at work 2. **Draw Pad** Dial your own phone # 3. **Directory** Call Matt Plumb’s Cell phone 4. **Alphabet Ribbon** Dial 801-555-1234 5. **Directory** Call William Dunn at work 6. **Draw Pad** Dial your own phone # 7. **Draw Pad** Call Opal Woods 8. **Alphabet Ribbon** Dial 801-555-1234 9. **Alphabet Ribbon** Call Frank Fontain at work 10. **Directory** Call Brittany Sanders’ Cell Phone | 1. **Alphabet Ribbon** Dial your own phone # 2. **Draw Pad** Call Helen Harris 3. **Alphabet Ribbon** Call Jessica Day 4. **Draw Pad** Dial 801-555-1234 5. **Directory** Call Ethan Hawke 6. **Draw Pad** Dial your own phone # 7. **Draw Pad** Call Lisa Hamilton 8. **Alphabet Ribbon** Dial 801-555-1234 9. **Directory** Call Brittany Sanders’ Cell Phone 10. **Draw Pad** Dial your own phone # |
| --- | --- |

**AA. Audi Q7 3.0 T Quattro Premium Plus *Send New Message* Tasks**

- **Participant sends a new text message to a phone contact using:**
  - **Draw Pad**
  - **Alphabet Ribbon**
- **Task is complete once message has been sent.**

| 1. Send “Please Call Back!” to Carly Duncan **DRAW PAD** 2. Send “No” to Albert Fink **ALPHABET RIBBON** 3. Send “Congratulations!”to Jessica Day **DRAW PAD** 4. Send “I’m in the car, and I’ll be late because of traffic” to Eleanor Lamb **ALPHABET RIBBON** 5. Send “Conference Cancelled” to William Dunn **ALPHABET RIBBON** 6. Send “See you later” Andrew Ryan **DRAW PAD** 7. Send “When & Where shall we meet?” to David Jones’ Cell **ALPHABET RIBBON** 8. Send “Ok, thanks for the info.” to Ana Pearl **DRAW PAD** 9. Send “I’m in the car, I’ll call later.” to Lisa Hamilton **DRAW PAD** 10. Send “Ok” to Opal Woods **ALPHABET RIBBON** | 1. Send “Please Call Back!” to Brittany Sanders’ Work **ALPHABET RIBBON** 2. Send “No” to Quin Brown **DRAW PAD** 3. Send “Congratulations!” to Helen Harris **ALPHABET RIBBON** 4. Send “I’m in the car, and I’ll be late because of traffic” to Robert Lutece **DRAW PAD** 5. Send “Conference Cancelled” to Ethan Hawke **ALPHABET RIBBON** 6. Send “See you later” to David Jones’ Work **DRAW PAD** 7. Send “When & Where shall we meet?” to Matt Plumb’s Cell **DRAW PAD** 8. Send “Ok, thanks for the info.” to Phil Dunphee **ALPHABET RIBBON** 9. Send “I’m in the car, I’ll call later.” to Helen Harris **DRAW PAD** 10. Send “Ok” to Zane Thompson **ALPHABET RIBBON** |
| --- | --- |

**BB. Audi Q7 3.0 T Quattro Premium Plus Navigation Tasks**

- **Participant sets the destination to a point of interest that best fits the task goal using:**
  - **Rotary Wheel**
  - **Draw Pad**
  - **Alphabet Ribbon**
- **Participant cancels the route before the task is considered to be complete.**

| 1. **Categories Menu** Bank 2. **Draw Pad** Zuppa’s Cafe 3. **Alphabet Ribbon** Gas Station 4. **Categories Menu** Hotel 5. **Draw Pad** Museum 6. **Alphabet Ribbon** ATM 7. **Categories Menu** Greek Restaurant 8. **Draw Pad** NoBrow Coffee 9. **Alphabet Ribbon** Fast Food 10. **Draw Pad** Library | 1. **Categories Menu** Movie Theater 2. **Alphabet Ribbon** Whole Foods 3. **Draw Pad** Post Office 4. **Categories Menu** Shopping Center 5. **Alphabet Ribbon** Hospital 6. **Draw Pad** Rest Area 7. **Categories Menu** Winery 8. **Alphabet Ribbon** Pizza 9. **Draw Pad** Apple Store 10. **Categories Menu** Brewery |
| --- | --- |

Table 1. A listing of the IVIS tasks and modes of interaction tested in each vehicle. Specific task lists were developed to test the different vehicles because they supported different combinations features and functions. In the table, the letter combinations (e.g., A-BB) refer to specific task set instructions that are described in detail in Appendix 1. Column headers refer to the different task by modality combinations. AE CS refers to audio entertainment performed using the center stack. AE AV refers to audio entertainment performed using the auditory vocal mode of interaction. AE CC refers to audio entertainment using the center console. CD CS refers to calling and dialing performed using the center stack. CD AV refers to calling and dialing performed using the auditory vocal mode of interaction. CD CC refers to calling and dialing using the center console. TXT CS refers to text messaging performed using the center stack. TXT AV refers to text messaging performed using the auditory vocal mode of interaction. TXT CC refers to text messaging using the center console. NAV CS refers to navigation performed using the center stack. NAV AV refers to navigation performed using the auditory vocal mode of interaction. NAV CC refers to navigation using the center console.

| **Vehicle** | **Condition** | | | | | | | | | | | |
| --- | --- | --- | --- | --- | --- | --- | --- | --- | --- | --- | --- | --- |
|  | **AE CS** | **AE AV** | **AE CC** | **CD CS** | **CD AV** | **CD CC** | **TXT CS** | **TXT AV** | **TXT CC** | **NAV CS** | **NAV AV** | **NAV CC** |
| Audi Q7 3.0T Quattro Premium Plus |  | **O_1_** | **Y** |  | **R** | **Z** |  | **W** | **AA** |  | **X_1_** | **BB** |
| BMW 430I |  | **A** | **A** |  | **E** | **E** |  |  |  |  | **J** | **J** |
| Buick Enclave | **A** | **A** |  | **E** | **E** |  | **G** | **I** |  |  |  |  |
| Cadillac XT5 Luxury | **M** | **M** |  | **R** | **R** |  |  |  |  |  |  |  |
| Chevrolet Equinox LT | **M** | **M** |  | **S** | **R** |  | **U** |  |  |  |  |  |
| Chevrolet Silverado LT | **A** | **A** |  | **E** | **E** |  | **G** |  |  |  |  |  |
| Chevrolet Traverse LT | **M** |  |  |  | **R** |  |  |  |  |  |  |  |
| Chrysler 300 C | **A** | **A** |  | **E** | **E** |  | **G** | **H** |  | **J** | **J** |  |
| Dodge Durango GT | **A** | **A** |  | **E** | **E** |  | **G** | **H** |  | **J** | **J** |  |
| Ford F250 XLT | **O_1_** | **M** |  | **T** | **R** |  | **W** | **W** |  |  |  |  |
| Ford Fusion Titanium | **M** | **M** |  | **R** | **R** |  | **U** | **U** |  |  |  |  |
| Ford Mustang GT | **A** | **A** |  | **E** | **E** |  | **G** | **I** |  | **J** | **J** |  |
| GMC Yukon SLT | **C, A*** | **A** |  | **E** | **E** |  | **H** |  |  | **L** | **J** |  |
| Honda Civic Touring | **A** | **A** |  | **F** | **E** |  | **H** |  |  | **J** | **J** |  |
| Honda Ridgeline RTL-E | **A** | **A** |  | **F** | **E** |  | **H** |  |  | **J** | **J** |  |
| Hyundai Santa Fe Sport | **M** | **Q** |  | **R** | **R** |  |  |  |  |  |  |  |
| Hyundai Sonata Base | **A** | **B** |  | **F** | **E** |  |  |  |  |  |  |  |
| Infiniti Q50 3.0T Premium | **O_1_** | **O_1_** | **O_2_** | **T** | **R** | **T** | **U** | **V** | **U** |  |  |  |
| Jeep Compass Sport | **P** | **Q** |  |  | **R** |  |  | **V** |  |  |  |  |
| Jeep Grand Cherokee Limited | **O_1_** | **N** |  | **R** | **R** |  | **W** | **V** |  | **X_1_** | **X_1_** |  |
| Kia Optima LX | **A** | **B** |  | **E** | **E** |  |  |  |  |  |  |  |
| Kia Sorento LX | **M** | **Q** |  | **R** | **R** |  |  |  |  |  |  |  |
| Kia Sportage LX | **A** | **B** |  | **F** | **E** |  |  |  |  |  |  |  |
| Land Rover Range Rover Sport | **A** | **A** |  | **E** | **E** |  |  |  |  | **J** |  |  |
| Lincoln MKC Premiere 2.0L | **O_1_** | **O_1_** |  | **R** | **R** |  | **W** | **U** |  |  |  |  |
| Mazda3 Touring |  | **M** | **M** |  | **R** | **R** |  | **U** | **U** |  |  |  |
| Mercedes C300 |  |  | **A** |  |  | **E** |  |  | **G** |  |  |  |
|  | *Manual (C), Touchscreen (A) | | | | | | | | | | | |
| **Vehicle (continued)** | **Condition (continued)** | | | | | | | | | | | |
|  | **AE CS** | **AE AV** | **AE CC** | **CD CS** | **CD AV** | **CD CC** | **TXT CS** | **TXT AV** | **TXT CC** | **NAV CS** | **NAV AV** | **NAV CC** |
| Nissan Armada SV | **A** |  |  | **F** | **E** |  |  |  |  | **K** | **K** |  |
| Nissan Maxima SV | **M** | **M** | **O_2_** | **T** | **R** | **T** | **U** | **V** | **U** | **X_2_** | **X_2_** | **X_2_** |
| Nissan Rogue SV | **A** |  |  |  | **E** |  |  | **H** |  |  |  |  |
| Ram 1500 Express | **M** | **N** |  | **R** | **R** |  |  | **V** |  |  |  |  |
| Ram 1500 Laramie | **A** | **A** |  | **E** | **E** |  |  | **I** |  |  |  |  |
| Subaru Crosstrek Premium | **A** | **B** |  | **F** | **E** |  |  |  |  |  |  |  |
| Tesla Model S 75 | **A** | **D** |  | **E** | **E** |  |  |  |  | **J** | **J** |  |
| Toyota Camry SE | **M** |  |  | **S** | **R** |  | **U** | **V** |  |  |  |  |
| Toyota Corolla SE | **O_1_** |  |  | **S** | **R** |  | **U** | **V** |  |  |  |  |
| Toyota RAV4 XLE | **O_1_** | **O_1_** |  | **S** | **R** |  | **U** | **V** |  |  |  |  |
| Toyota Sienna XLE | **O_1_** | **O_1_** |  | **S** | **R** |  | **U** | **V** |  |  |  |  |
| Volkswagen Jetta S | **A** |  |  | **E** |  |  |  |  |  |  |  |  |
| Volvo XC60 T5. Inscription | **M** | **M** |  | **R** | **R** |  | **W** | **W** |  | **X_2_** | **X_2_** |  |

**Table 2.** A listing of IVIS tasks performed using the different modes of interaction. Note that the vehicles tested support different combinations of tasks and modes of interaction (see Table 1 for details).

|  | **Center Stack** | **Auditory Vocal** | **Center Console** |
| --- | --- | --- | --- |
| **Audio Entertainment** | a) Tune the radio to <*station #>*  b) Play <*artist name/song title/genre type*>  c) Change the audio source to <*iPod/AM/FM/XM/Bluetooth*> | a) Tune the radio to <*station #>*  b) Play <*artist name/song title/genre type*>  c) Change the audio source to <*iPod/AM/FM/XM/Bluetooth*> | a) Tune the radio to <*station #>*  b) Play <*artist name/song title/genre type*>  c) Change the audio source to <*iPod/AM/FM/XM/Bluetooth*> |
| **Calling and Dialing** | a) Call <*Contact name at work/on mobile>*  b) Dial *<participant’s own phone number*  c) Dial *<801-555-1234>* | a) Call <*Contact name at work/on mobile>*  b) Dial *<participant’s own phone number>*  c) Dial *<801-555-1234>* | a) Call <*Contact name at work/on mobile>*  b) Dial *<participant’s own phone number>*  c) Dial *<801-555-1234>* |
| **Text Messaging** | a) Reply to a text message in the inbox with <*a predetermined message*>  b) NA  c) Listen to a text message in the inbox from <*Contact Name>* | a) Reply to a text message in the inbox with <*a predetermined message*>  b) Send a new text message to <*Contact Name>* that says <*a pre-determined message*>  c) Listen to a text message <*text message>* | a) Reply to a text message in the inbox with <*a predetermined message*>  b) Send a new text message to <*Contact Name*> that says <*a pre-determined message*>  c) NA |
| **Navigation** | a) Navigate to <*point of interest>* | a) Navigate to *<point of interest>* | a) Navigate to *<point of interest>* |
